# Anthranilic acid from *Ralstonia solanacearum* plays dual roles in intraspecies signalling and inter-kingdom communication

**DOI:** 10.1038/s41396-020-0682-7

**Published:** 2020-05-26

**Authors:** Shihao Song, Wenfang Yin, Xiuyun Sun, Binbin Cui, Lei Huang, Peng Li, Liang Yang, Jianuan Zhou, Yinyue Deng

**Affiliations:** 1 College of Agriculture, South China Agricultural University, Guangzhou, 510642 China; 2 School of Pharmaceutical Sciences (Shenzhen), Sun Yat-sen University, Guangzhou, 510275 China; 3 Ministry of Education Key Laboratory for Ecology of Tropical Islands, College of Life Sciences, Hainan Normal University, Haikou, 571158 China; 4 School of Medicine, Southern University of Science and Technology, Shenzhen, 518055 China

**Keywords:** Environmental microbiology, Microbial communities, Biofilms

## Abstract

Quorum sensing (QS) signals are widely utilized by bacteria to regulate biological functions in response to cell population density. Previous studies have demonstrated that *Ralstonia solanacearum* employs two different types of QS systems. We report here that anthranilic acid controls important biological functions and the production of QS signals in *R. solanacearum*. It was demonstrated that the biosynthesis of anthranilic acid is mainly performed by TrpEG. The accumulation of anthranilic acid and the transcription of *trpEG* occur in a cell density-dependent manner in *R. solanacearum*. Both the anthranilic acid and TrpEG homologues are conserved in various bacterial species. Moreover, we show that *Sporisorium scitamineum* sexual mating and hypha formation are strongly inhibited by the addition of exogenous anthranilic acid. Our results suggest that anthranilic acid is important for the physiology of bacteria in addition to its role in inter-kingdom communication.

## Introduction

Quorum sensing (QS) is a cell-to-cell communication mechanism used by many bacterial species to coordinate communal behaviour in response to cell density to regulate phenomena such as virulence, biofilm formation, motility and antibiotic production [1–3]. When a minimal threshold concentration of a QS signal is reached, it will cause transcriptional activation and repression of a large regulon of target genes. The first well-characterized QS system utilized by many Gram-negative bacteria is mediated by N-acyl-L-homoserine lactones (AHLs) as extracellular signalling molecules [2, 4–6]. In addition to AHL family signals, there are many other kinds of QS signals, such as diffusible signal factor (DSF)-type signals [3, 7], autoinducer 2 [8, 9], methyl 3-hydroxypalmitate (3-OH PAME) [10], 2-heptyl-3-hydroxy-4(1 H)-quinolone (PQS) [11], 2-heptyl-4-quinolone (HHQ) [12], AI-3 [13], bradyoxetin [14], and diketopiperazines [15].

*Ralstonia solanacearum* is a soil-borne bacterium that causes a lethal disease known as “bacterial wilt” in more than 200 plant species worldwide [16–18]. Previous studies showed that methyl 3-OH PAME is a QS signal that plays a major role in the global regulation of *R. solanacearum* virulence factors, including extracellular polysaccharide (EPS), cell wall-degrading enzymes and motility [10, 16, 19]. It was later found that methyl 3-hydroxymyristate (3-OH MAME) was synthesized by several *R. solanacearum* strains [19]. 3-OH PAME or 3-OH MAME is synthesized by PhcB, which is a methyltransferase. When the QS signals reach a threshold level, it activates the histidine kinase PhcS to phosphorylate the response regulator PhcR and increase the expression levels of PhcA, which is a LysR-type transcriptional regulator that plays a central role in the *phc* system as a global regulator [10, 20, 21]. In addition, it was found that *R. solanacearum* possesses a *solIR* QS system, which is a *luxI/luxR* homologue that is upregulated by the *phc* system [10, 19, 22]. The abolition of *solIR* eliminates the synthesis of AHL signals but does not affect virulence factor production in *R. solanacearum* AW1 strain  [22].

Microbial crosstalk between bacteria and fungi commonly exists in many ecological settings. Small molecule signals are increasingly recognized as important interspecies or inter-kingdom communication signals in microbial communities [23]. Ralsolamycin from *R. solanacearum* has been identified as an inducer of chlamydospore formation in fungi such as *Ascomycetes*, *Basidiomycetes* and *Zygomycetes* [24]. The biosynthesis of ralsolamycin in *R. solanacearum* is modulated by the two nonribosomal peptide synthetases, RmyA and RmyB [25]. Interestingly, our previous study revealed that the expression of *rmyAB* was controlled by the *phcBSR* QS system [26], suggesting that QS plays an important role in inter-kingdom communication between *R. solanacearum* and fungi. In this study, we showed that anthranilic acid produced by TrpEG in *R. solanacearum* inhibits sexual mating and hypha formation in *Sporisorium scitamineum*. The inactivation of the anthranilate synthase gene, *trpEG*, disrupts biofilm formation, motility activity, virulence factor production and pathogenicity in *R. solanacearum*. The addition of exogenous anthranilic acid restored these phenotypes of the *trpEG* deletion mutant strain. Furthermore, it was indicated that anthranilic acid controls the expression levels of many genes in *R. solanacearum*. As *R. solanacearum* and *S. scitamineum* are frequently encountered plant pathogens, our data suggest that in addition to the important role of anthranilic acid in the physiology of *R. solanacearum*, it also engages in an antagonistic interaction whose importance to microbial ecology and pathogenesis is now becoming evident.

## Materials and methods

### Bacterial strains and growth conditions

The bacterial strains and plasmids used in this work are listed in Supplementary Table 1. *R. solanacearum* GMI1000 (ATCCBAA-1114) was obtained from the American Type Culture Collection and maintained at 28 °C in 2,3,5-triphenyltetrazolium chloride medium (TTC medium) containing 1% tryptone, 0.1% casamino acids, 0.5% glucose, and 0.005% 2,3,5-triphenyltetrazolium chloride [27]. Luria–Bertani medium (1 l contains 10 g tryptone, 5 g yeast extract and 10 g NaCl, pH 7.0) was used to maintain *Escherichia coli*. *S. scitamineum* was cultured in YePS agar medium (10 g yeast extract, 20 g peptone, 20 g sugar, 20 g agar per litre) [28]. The following antibiotics were added when necessary: ampicillin and kanamycin at 100 µg ml^−1^; tetracycline, 10 µg ml^−1^; and gentamicin, 10 µg ml^−1^. Bacterial growth was determined by measuring the optical density at a wavelength of 600 nm.

### Purification and structural analysis of anthranilic acid from *R. solanacearum*

*R. solanacearum* GMI1000 cells were cultured in TTC medium for 48 h, and the supernatant was extracted with an equal volume of ethyl acetate. Following evaporation of the ethyl acetate, the residue was dissolved in methanol and subjected to HPLC analysis in a C18 reverse-phase column (XBridge, 10 μm, 19 mm × 250 mm, Waters) and eluted with methanol-water (from 35:65 to 100:0 v/v) at a flow rate of 7 ml/min. The active fractions were detected, concentrated, purified by HPLC using a semi-preparative C18 reverse-phase column (Gemini-NX, 5 μm, 10.0 mm × 250 mm, Phenomenex) and eluted with methanol-water (from 35:65 to 100:0 v/v) at a flow rate of 3 ml/min. Peaks were monitored using a ultraviolet (UV) detector at 210 nm and were collected and assayed. The experiment was performed according to the previous method [27].

The ^1^H, ^13^C, and DEPT135 nuclear magnetic resonance (NMR) spectra in CD_3_OD solution were obtained using a Bruker AV-500 (Bruker Instrument, Inc., Zurich, Switzerland) spectrometer operating at 500 MHz for ^1^H or 125 MHz for ^13^C. High-resolution electrospray ionization mass spectrometry (HR-ESI-MS) was performed on a Waters Q-Tof Premier high-resolution mass spectrometer (Waters, Milford- MA, USA) [27].

### Analysis of sexual mating and filamentation in *S. scitamineum*

The haploid *S. scitamineum* MAT‐1 and MAT‐2 cell lines expressing green fluorescent protein (GFP) and red fluorescent protein (RFP), respectively, were used for the bioassays of the sexual mating and filamentation of *S. scitamineum*. YePS plates were cut into separated slices (0.6 × 6 cm). Ten microlitres of anthranilic acid or other chemicals were spotted at the end of the slices in the agar plate at 0, 10, 25, 50 or 100 μM. Thereafter, the MAT‐1 and MAT‐2 cell line mixture (1 μl, OD_600_ ≈ 1.5) was spotted on the slices. The plates were incubated at 28 °C for 48 h until white hyphae grew in the negative control. For microscopic analyses, *S. scitamineum* cells were cultured in YePS liquid medium for 24 h. The cells were then transferred to YePS plates with or without the addition of anthranilic acid for 24 h, then washed off with sterile water and observed and imaged using a Leica DMi8 microscope equipped with a Leica DFC450 C camera (Leica, Wetzlar, Germany). Standard filter sets of GFP, RFP and differential interference contrast were used [29]. The bioassay was repeated at least three times.

### Construction of reporter strains and measurement of β-galactosidase activity

The *trpEG* promoter was amplified using the p*trpEG*-F and p*trpEG*-R primer pair listed in Supplementary Table 2 and then purified prior to ligation with the expression vector pME2-*lacZ*, which was digested with the same enzymes. The *trpEG* reporter was introduced into the *R. solanacearum* GMI1000 and *trpEG* mutant strains by electroporation. Transconjugants were then selected on TTC agar plates supplemented with tetracycline and X-gal. β-galactosidase activities were assayed following the methods described previously [30]. Bacteria were cultured at 28 °C, and the cells were harvested to measure β-galactosidase activities.

### Construction of in-frame deletion mutants and complementation

*R. solanacearum* GMI1000 was used as the parental strain for the generation of in-frame deletion mutants following the methods described previously [31]. The primers used to generate the upstream and downstream regions flanking *trpEG* are listed in Supplementary Table 2. For complementation analysis, the coding regions of *trpEG* were amplified and cloned into the pLAFR3 plasmid. The resulting constructs were introduced into *R. solanacearum* GMI1000 deletion mutants using electroporation.

### Virulence assays in tomato plants

The analysis of the virulence of the *R. solanacearum* WT, ΔtrpEG and complementation strains was performed in an AIRKINS greenhouse (28 °C, light 16 h and dark 8 h). A mixture including field soil, sand, and compost (1.25:1.25:0.5) was prepared and autoclaved at 121 °C for 20 min. Tomato seeds (Jinfeng 1) were surface-sterilized in 2% NaClO for 3 min and 75% ethanol for 2 min, rinsed 3 times in sterile water, and then planted in soil. *R. solanacearum* cells were grown in TTC medium to an OD_600_ = 1.0. Aliquots of 5 ml of the *R. solanacearum* WT, ∆trpEG, and ∆trpEG(trpEG) fermentation broth were inoculated onto the tomato seedlings. Each treatment included 25 replicates [27].

### Analysis of *R. solanacearum* cell numbers in plants

One gram samples of plant roots and stems were collected and milled in 9 ml of sterilized water for 20 min. The suspensions were serially diluted and spread on TTC plates. The plates were incubated at 28 °C for 48 h. Then, the numbers of *R. solanacearum* cells were counted [27].

### Statistical analysis

Statistical analyses were performed with Prism8 software (GraphPad). The data are presented as the means ± standard deviations of three independent experiments. Statistical significance was indicated as follows: *p < 0.05; **p < 0.01; ***p < 0.001 (unpaired *t* test). All results were calculated from the means of at least three replicates.

## Results

### *R. solanacearum* inhibits the sexual mating and morphological transition of *S. scitamineum*

**Fig. 1:**
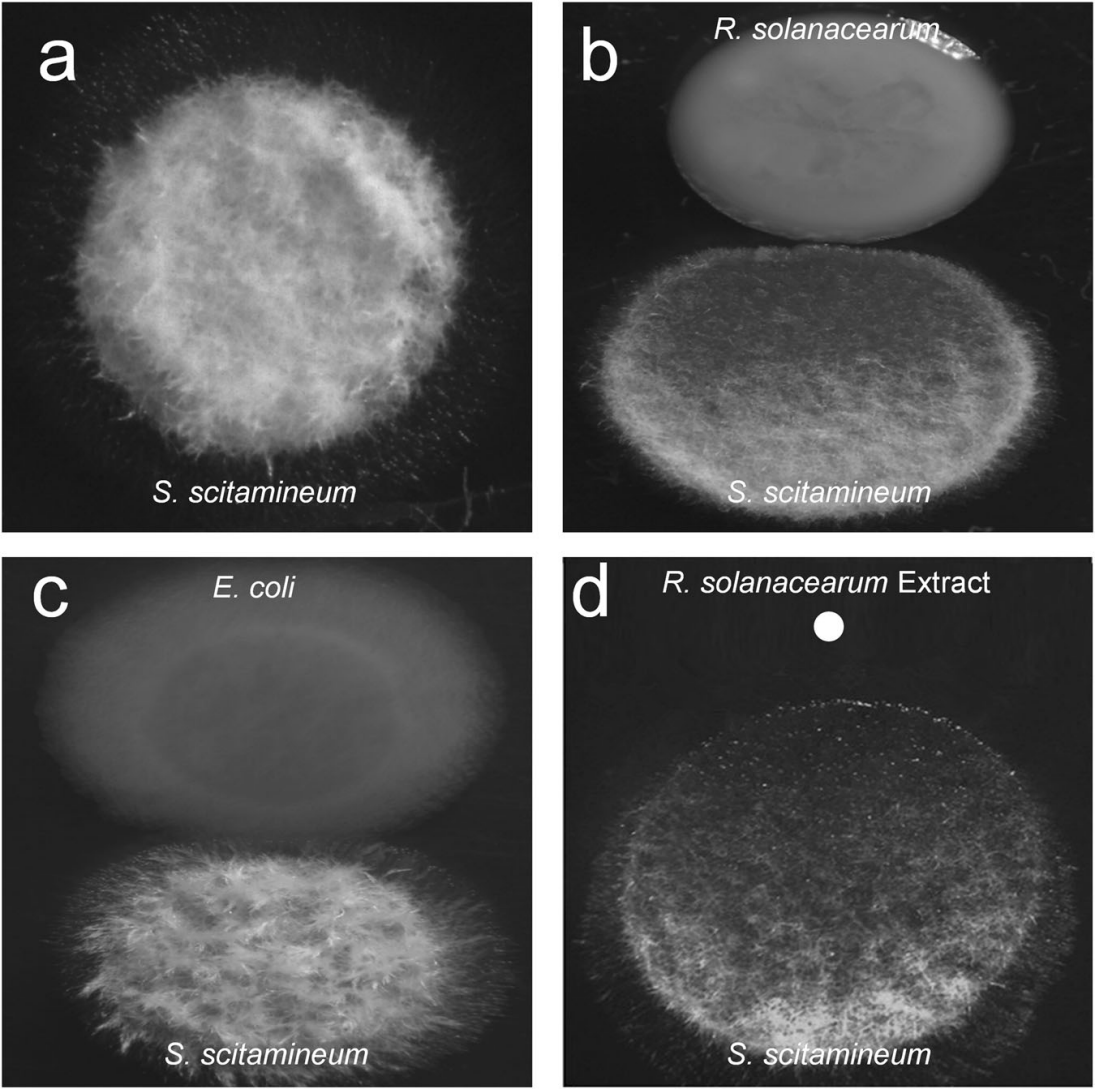
Effect of *R. solanacearum* on  *S. scitamineum* sexual mating and morphological transition. The hypha formation of *S. scitamineum* was analysed when it was grown in a plate in the absence (**a**) or presence of *R. solanacearum* colonies (**b**), *E. coli* colonies (**c**) and the ethyl acetate extract of *R. solanacearum* (**d**).

A previous study showed that *R. solanacearum* produces ralsolamycin to induce chlamydospore formation in fungi [24]. As both *R. solanacearum* and *S. scitamineum* are plant pathogens, we investigated whether competition exists between the two pathogens. When they were grown together in the plate containing YePS medium, we found that *R. solanacearum* GMI1000 exhibited obvious inhibitory activity toward sexual mating and hypha formation in *S. scitamineum*, while *S. scitamineum* produced hyphae well when it was inoculated either alone or with *E. coli* DH5α (Fig. 1a–c). We then extracted small molecular weight compounds from the liquid culture of *R. solanacearum* using ethyl acetate and found that the ethyl acetate extract exerted a similar inhibitory effect on sexual mating and hypha formation in *S. scitamineum* (Fig. 1d), suggesting that *R. solanacearum* produces some compounds with inhibitory activity against *S. scitamineum* sexual mating and morphological transition.

### The active component of *R. solanacearum* is anthranilic acid

**Fig. 2:**
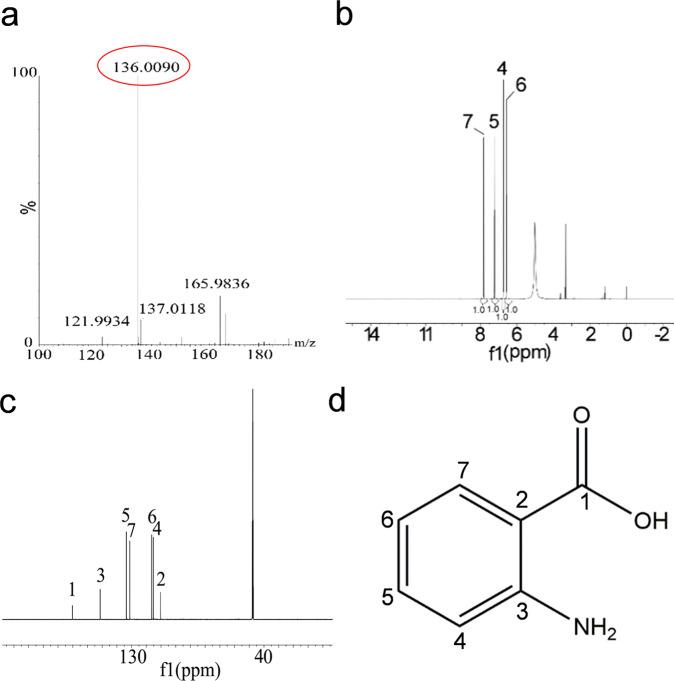
Structural characterization of anthranilic acid. **a** ESI-MS spectra of anthranilic acid. **b** ^1^H NMR spectra of anthranilic acid. **c** ^13^C NMR spectra of anthranilic acid. **d** Chemical structure of anthranilic acid.

To identify the active component of *R. solanacearum* that inhibits the sexual mating and morphological transition of *S. scitamineum*, we isolated and purified the active fractions from 100 L *R. solanacearum* GMI1000 culture supernatants using High Performance Liquid Chromatography (HPLC). Approximately 93.4 mg of the purified compound showing activity that inhibits the sexual mating of *S. scitamineum* was obtained. HR-ESI-MS spectrometry analysis of the active compound revealed a molecular ion [M-H]^-^ with an m/z ratio of 136.0405, matching with a molecular formula of C_7_H_7_NO_2_. It was observed that there were four protons in the aromatic region in the ^1^H spectrum, in addition to acidic protons deuterated by the D4-MeOH solvent. While carbons from seven distinctive chemical environments were identified from the ^13^C spectrum, a comparison with Distortionless Enhancement by Polarization Transfer (DEPT) 135 showed the likely existence of one carbonyl C along with two extra Cs and four CHs in the aromatic region. The results were in agreement with the literature [32] (Supplementary Table 3), which indicated that the active compound was anthranilic acid (Fig. 2, Supplementary Fig. 1).

### Anthranilic acid inhibits *S. scitamineum* sexual mating and morphological transition in a dose-dependent manner

**Fig. 3:**
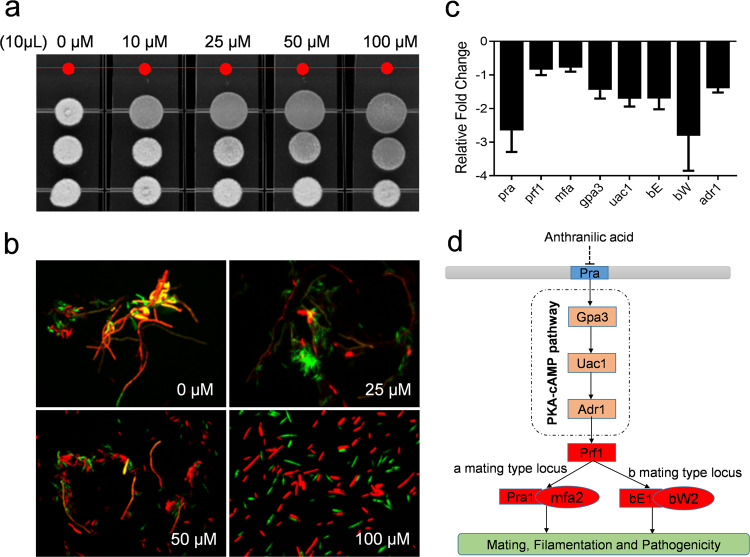
Effect of anthranilic acid on *S. scitamineum* sexual mating and morphological transition. The sexual mating and morphological transition of *S. scitamineum* were analysed when it was grown in a plate (**a**) and then observed in water under a fluorescence microscope (**b**). **c** Comparison of the relative fold-changes of regulator-encoding genes in *S. scitamineum* with and without the addition of anthranilic acid. **d** Schematic diagram of the signalling pathways that govern sexual mating and filamentation in *S. scitamineum* affected by anthranilic acid.

To determine whether the effects of anthranilic acid on *S. scitamineum* are specific and related to the dose, different concentrations of the standard anthranilic acid compound were assessed for their inhibitory activity against the sexual mating and morphological transition of *S. scitamineum* (Fig. 3). These effects were assayed using a mixture of haploid *S. scitamineum* cell lines. In its nonpathogenic form, this fungus grows as a budding saprophytic haploid. The fusion of compatible haploids to form an infectious dikaryon initiates the pathogenic cycle. Sexually compatible haploids fuse to form the filamentous dikaryon, and the dikaryon invades plants and grows within and between plant cells, triggering the formation of characteristic tumours [33–35]. It was shown that anthranilic acid had an obvious inhibitory effect on the sexual mating of *S. scitamineum* at a final concentration of 10 μM (Fig. 3a), and the observed inhibition increased with the addition of higher concentrations of anthranilic acid (Fig. 3a). We also used the haploid *S. scitamineum* MAT-1 and MAT-2 cell lines, which express GFP and RFP, respectively, for the analysis of sexual mating and hypha formation. We found that sexual mating and hyphal formation were almost completely inhibited when anthranilic acid was supplemented at a final concentration of 100 μM, while anthranilic acid did not affect the growth rate of *S. scitamineum* cells at this concentration (Fig. 3b, Supplementary Fig. 2). We also tested the effects of benzoic acid and one isomer of anthranilic acid, P-aminobenzoic acid, on the sexual mating and hypha formation of *S. scitamineum* and found that they exhibited no inhibitory activity (Supplementary Fig. 3).

### Anthranilic acid inhibits *S. scitamineum* sexual mating and filamentation by interfering with the PKA-cAMP pathway

To explore a working model of the effect of anthranilic acid on the sexual mating and filamentation of *S. scitamineum*, we continued to test whether anthranilic acid interfered with the signalling pathways involved sexual mating and hyphal development. In *S. scitamineum*, cell fusion is controlled by a pheromone/receptor system encoded by the “a” locus, while the establishment of hyphae is dependent on an active heterodimer of the homeodomain transcription factors expressed from the multiallelic “b” locus. After cell fusion, the merging of the cytoplasm of compatible cells leads to the formation of the bE/bW transcription factor to trigger hypha formation on the surface of the plant epidermis [36]. The mitogen-activated protein kinase and cyclic AMP and protein kinase A system pathways are necessary for sensing pheromone and environmental signals. The two pathways are involved in the transcriptional and post-translational activation of the transcription factor Prf1 (Pheromone-responsive factor 1). Prf1 is a central regulatory factor that controls mating and virulence [37–42]. Quantitative Real-time Polymerase Chain Reaction (qRT-PCR) analysis showed that the addition of exogenous anthranilic acid inhibited the expression levels of *pra, gpa3, uac1, adr1, prf1, mfa, bE* and *bW*, which encode the components of the PKA-cAMP pathway (Fig. 3c). Collectively, these results demonstrated that anthranilic acid inhibited expression levels of the components of PKA-cAMP pathway to interfere with *S. scitamineum* sexual mating and filamentation (Fig. 3d).

### TrpEG is responsible for anthranilic acid biosynthesis in *R. solanacearum*

**Fig. 4:**
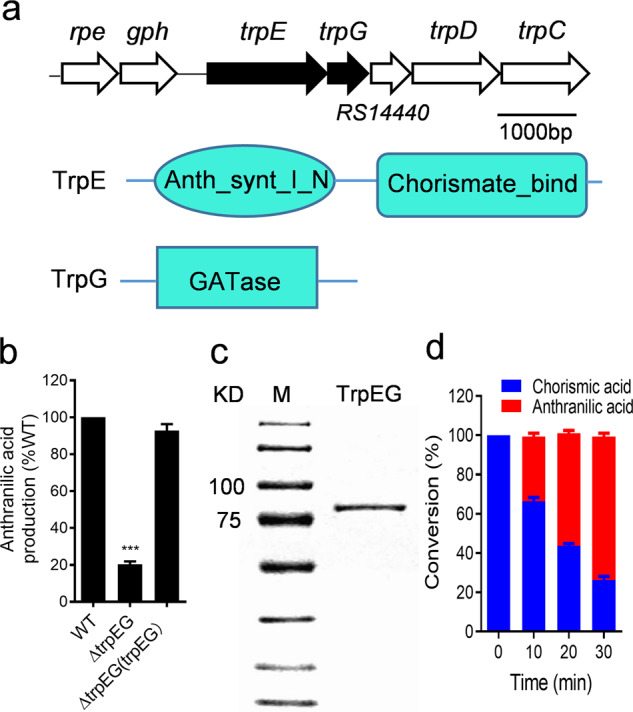
Analysis of the enzyme activity of TrpEG in the synthesis of anthranilic acid. **a** Genomic organization of the *trpEG* region in *R. solanacearum* GMI1000 (top). Domain structure analysis of TrpE (middle) and TrpG (bottom). **b** Detection of anthranilic acid production via the LC-MS assay. **c** SDS-PAGE analysis of the TrpEG protein. **d** Analysis of the production of anthranilic acid by TrpEG to catalyse chorismic acid transformation. The data are means ± standard deviations of three independent experiments. ***p < 0.001 (unpaired *t* test).

In *Pseudomonas aeruginosa*, the enzymes encoded by *trpEG* and *phnAB* are the key enzymes involved in the biosynthesis of anthranilic acid [43]. There is an alternative biosynthesis pathway for anthranilic acid production in which the *kynA*, *kynB*, and *kynU* genes encode tryptophan 2,3-dioxygenase, kynurenine formamidase, and kynureninase, which decompose tryptophan to anthranilic acid [44]. To identify the genes responsible for anthranilic acid biosynthesis, *trpEG* homologues were first searched in the genome sequence of *R. solanacearum* GMI1000 and identified as RS_RS14430 and RS_RS14435 by using the National Center for Biotechnology Information Basic Local Alignment Search Tool (BLAST) program [45] (Fig. 4a). The deletion of *RS_RS14430* and *RS_RS14435* (*trpEG*) together almost completely abolished anthranilic acid production (Fig. 4b) but only resulted in a slight decrease in the growth rate of the bacterial cells in both rich and poor nutrient media (Supplementary Fig. 4). Interestingly, different from the *trpE* mutant of *Pseudomonas aeruginosa*, which did not grow in the minimal medium [46], the *trpEG* mutant of *R. solanacearum* GMI1000 grew well in MP minimal medium with a slight decrease of growth rate compared with that of the wild-type strain (Supplementary Fig. 4). We also identified the homologues of *kynA*, *kynB*, and *kynU* in *R. solanacearum* GMI1000. The deletion of the three genes caused only a slight reduction of anthranilic acid production (Supplementary Fig. 5), but double deletion of the *trpEG* and *kynAUB* genes abolished anthranilic acid production completely (Supplementary Fig. 5). These findings indicated that TrpEG was the most important synthase for the biosynthesis of anthranilic acid in *R. solanacearum*.

To further confirm the observed enzymatic activity in vitro, the fusion protein TrpEG, which contains 698 amino acids and has a calculated molecular weight of 77.33 kDa, was purified using affinity chromatography (Fig. 4c). In vitro enzyme activity analysis showed that TrpEG directly catalysed the transformation of chorismic acid to anthranilic acid (Fig. 4d; Supplementary Fig. 6).

### The production of anthranilic acid is cell density dependent

**Fig. 5:**
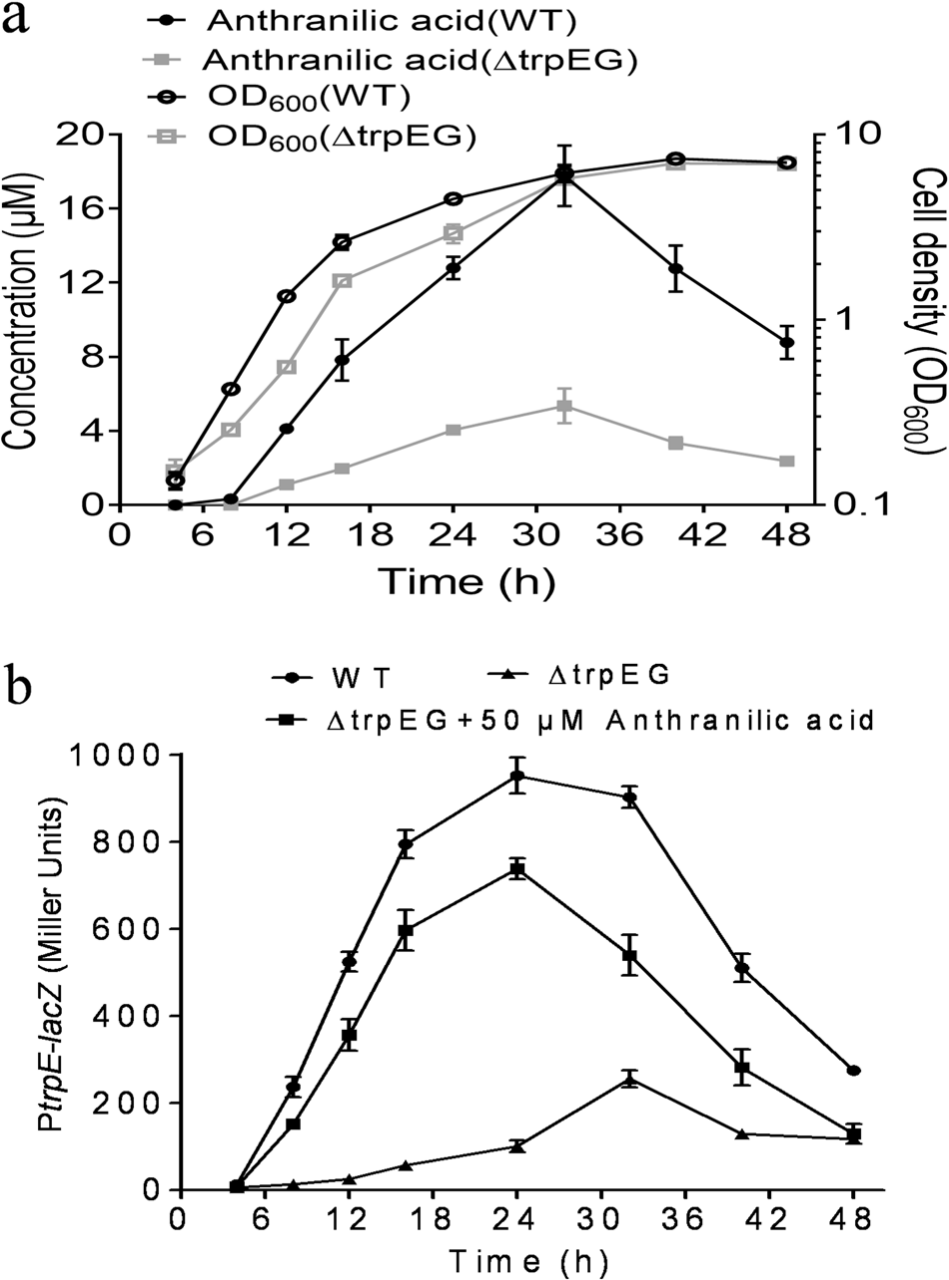
Analysis of anthranilic acid production and *trpEG* transcriptional expression. **a** Time-course analysis of anthranilic acid accumulation in the *R. solanacearum* wild-type strain (●) and the *trpEG* deletion mutant strain (■) and *R. solanacearum* cell growth in the wild-type strain (○) and the *trpEG* deletion mutant strain (□) in liquid medium. **b** β-Galactosidase activity of a *trpEG*-*lacZ* transcriptional fusion in the *R. solanacearum* wild-type strain (●) and the *trpEG* deletion mutant strain (▲) and the *trpEG* deletion mutant strain supplemented with anthranilic acid (■). The *R. solanacearum* GMI1000 strains were inoculated in a 2 L flask containing 1 L TTC medium and cultured at 28 °C with shaking at 220 rpm. The experiment was started at an initial OD_600_ of 0.05. The data are means ± standard deviations of three independent experiments.

To investigate whether the biosynthesis of anthranilic acid is related to cell density, we first measured the time course of anthranilic acid production by determining anthranilic acid concentrations at various growth stages. The amount of anthranilic acid was detectable and was first measured at 8 h postinoculation according to the standard curve of anthranilic acid (Fig. 5a; Supplementary Fig. 7). After this time point, anthranilic acid concentrations increased sharply during the exponential growth phase and peaked at 32 h in the late exponential growth phase, followed by a significant decline in the anthranilic acid concentration (Fig. 5a).

We continued to investigate the transcriptional profile of *trpEG*, which controls anthranilic acid production. A 411-bp DNA sequence containing the *trpEG* promoter region was transcriptionally fused to the *lacZ* coding region and introduced into the wild-type and *trpEG* mutant strains. The transcriptional level of *trpEG* in the wild-type strain gradually increased and peaked at 24 h postinoculation, then decreased (Fig. 5b), which was well correlated with the anthranilic acid accumulation profile.

To test whether the transcriptional expression of *trpEG* is autoregulated by anthranilic acid, we compared the transcriptional profiles of *trpEG* in both the wild-type strain and the *trpEG* deletion mutant strain. The results showed that promoter activity in the mutant strain was decreased significantly from that in the wild-type background, and exogenous addition of anthranilic acid could almost restore the promoter activity in the *trpEG* deletion mutant strain to the levels in the wild-type strain, suggesting that the production of anthranilic acid may be autoregulated at the transcriptional level (Fig. 5b).

### Deletion of *trpEG* impairs biological functions in *R. solanacearum*

**Fig. 6:**
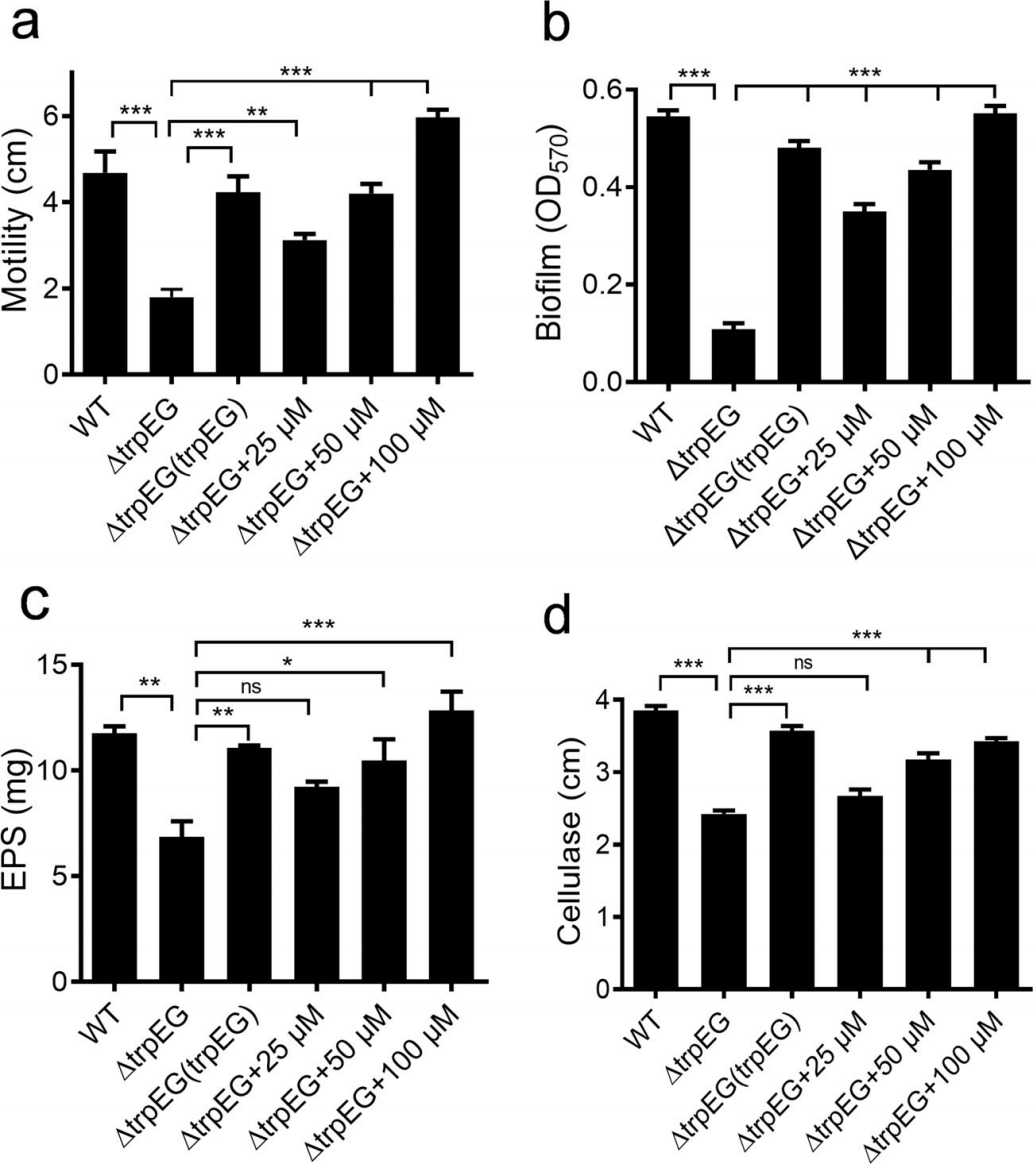
Effect of *trpEG* and the anthranilic acid compound on the QS-regulated phenotypes of* R. solanacearum*. Motility (**a**), biofilm formation (**b**), EPS production (**c**) and cellulase production (**d**) in *R. solanacearum* GMI1000  were analysed. The data are means ± standard deviations of three independent experiments. *p < 0.05; **p < 0.01; ***p < 0.001 (unpaired *t* test).

It was determined that anthranilic acid inhibits *S. scitamineum* sexual mating and morphological transition through inter-kingdom interference. We continued to study whether this compound plays a role in the regulation of biological functions in *R. solanacearum*. As the biosynthesis of anthranilic acid is mainly performed by TrpEG, we tested the phenotypes of biofilm formation, cellulase production, motility activity, and EPS production in an in-frame deletion mutant of *trpE*, *trpG*, and *trpEG*. It was shown that the deletion of *trpEG* had little effect on the growth of bacterial cells (Supplementary Fig. 4) but resulted in significant impairment of these phenotypes (Fig. 6). Interestingly, both the *in trans* expression of *trpEG* and the addition of exogenous anthranilic acid restored these phenotypes of the *trpEG* deletion mutant to wild-type strain levels (Fig. 6). As expected, the two single deletion mutants, *trpE* and *trpG*, showed the similar phenotypes as the *trpEG* double deletion mutant, and exogenous addition of anthranilic acid restored these phenotypes of the *trpE* deletion mutant and the *trpG* deletion mutant to the wild-type strain levels (Supplementary Figs. 8,9). We also found that deletion of *trpE*, *trpG* or *trpEG* abolished the inhibition on the sexual mating and hypha formation of *S. scitamineum* (Supplementary Fig. 10). Intriguingly, the deletion of *kynAUB* in both the wild-type and *trpEG* deletion mutant strains exhibited no effect on these phenotypes (Supplementary Fig. 11).

Quantitative Real-time PCR analysis showed that the mutation of *trpEG* caused a decrease in the expression levels of *phcA, phcB, solI, epsA, epsB, epsC, epsD, epsE* and *epsF* (Supplementary Fig. 12a). Among these genes, *phcA* and *phcB* encode the regulator and the signal synthase, respectively, while *epsA-F* are the target genes of the *phc* system. *soII* is the gene encoding the signal synthase of the *sol* system. Moreover, we also measured and compared the production of anthranilic acid, C8-AHL, C10-AHL and 3OH-MAME in the *trpEG* mutant, *phcB* mutant and wild-type strains. We found that the production of anthranilic acid, C8-AHL, C10-AHL and 3OH-MAME was reduced in the *trpEG* mutant strain, while only the production of C8-AHL, C10-AHL and 3OH-MAME was downregulated in the *phcB* mutant strain (Supplementary Fig. 12b). These results demonstrated that anthranilic acid/TrpEG positively regulated both the *phc* and *sol* QS systems in *R. solanacearum*.

### It is anthranilic acid rather than its metabolic products that controls the biological functions in *R. solanacearum*

In *Pseudomonas aeruginosa*, anthranilic acid is a precursor for the biosynthesis of three QS molecules, 2,4-dihydroxyquinoline (DHQ), HHQ and 2-heptyl-3-hydroxy-4-quinolone (PQS) [47]. To finally determine whether anthranilic acid serves as a precursor for the signals or acts itself, we used liquid chromatograph mass spectrometer (LC-MS) analysis to check whether the ethyl acetate extract of the *R. solanacearum* supernatant contained PQS, HHQ, or DHQ on the basis of comparison with the standard signals. It was revealed that there was no PQS, HHQ or DHQ in the extract (Supplementary Figs. 13–15). In addition, the addition of exogenous PQS, HHQ, or DHQ caused no restoration of the phenotypes of the *trpEG* mutant strain (Supplementary Fig. 16), suggesting that it is anthranilic acid controlling the biological functions in *R. solanacearum*.

We then measured the gene expression levels of *epsA* in the wild-type strain and the double deletion mutant strain ΔtrpEGΔkynAUB, which is anthranilic acid-deficient (Supplementary Fig. 5). As *epsA* was identified to be a target gene positively controlled by the anthranilic acid compound (Supplementary Fig. 12a), the result further confirmed that deletion of *trpEG* and *kynAUB* significantly decreased the promoter activity of *epsA* as expected (Supplementary Fig. 17). More interestingly, we found that exogenous addition of anthranilic acid at a final concentration of 1 μM obviously increased the expression level of *epsA* in the deletion mutant strain ΔtrpEGΔkynAUB; and addition of 20 μM anthranilic acid fully restored the expression level of *epsA* in the ΔtrpEGΔkynAUB deletion mutant strain to the wild-type strain level (Supplementary Fig. 17), suggesting that there is a minimal threshold concentration of the anthranilic acid compound in regulating target gene expression in *R. solanacearum*.

### Anthranilic acid contributes to *R. solanacearum* pathogenicity

**Fig. 7:**
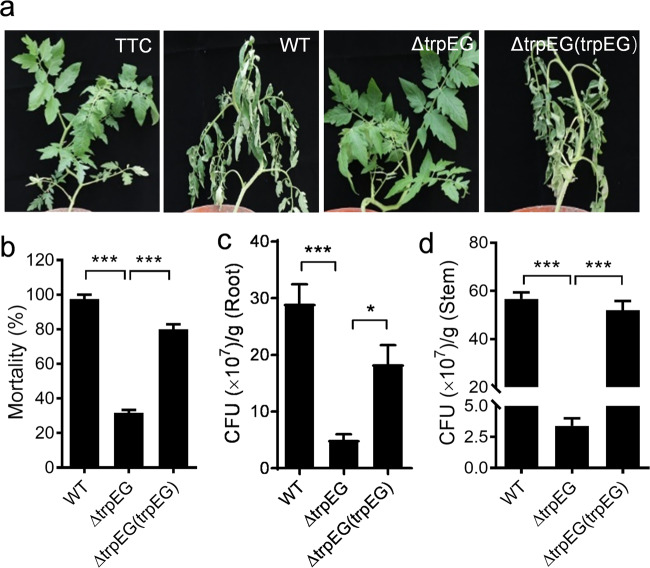
Influence of *trpEG* on the virulence of *R. solanacearum* in tomato plants. **a** Analysis of *R. solanacearum* virulence in tomato plants. **b** Mortality of tomato plants after infection with the *R. solanacearum* wild-type, ∆trpEG and ∆trpEG(trpEG) strains. CFUs of the *R. solanacearum* wild-type, ∆trpEG and ∆trpEG(trpEG) strains in the roots (**c**) and stems (**d**) of tomato plants. The data are means ± standard deviations of three independent experiments. *p < 0.05; **p < 0.01; ***p < 0.001 (unpaired *t* test).

To determine the role of anthranilic acid in *R. solanacearum* pathogenesis, the *R. solanacearum* GMI1000 wild-type strain, the *trpEG* mutant strain, and the complement strain were used to infect tomato plants. The plants treated with the *trpEG* mutant strain showed an obvious reduction of wilt symptoms compared with the plants infected by the wild-type and complement strains (Fig. 7a). Accordingly, the mortality of the plants treated with the *R. solanacearum* wild-type, *trpEG* mutant, and complement strains at 21 d was 97.5%, 31.7%, and 80%, respectively (Fig. 7b).

To further study the effect of anthranilic acid in the *R. solanacearum* infection process, we continued to quantify the colony-forming units (CFU) of *R. solanacearum* strains in both the roots and stems of the tomato plants. The CFUs recorded for the *R. solanacearum* wild-type, *trpEG* deletion mutant, and complement strains were 2.96 × 10^8^, 6.4 × 10^7^, and 1.98 × 10^8^ per gram of root tissue at 21 d postinoculation, respectively (Fig. 7c). A similar result was observed for tomato stems, in which the CFUs of the three strains were 5.67 × 10^8^, 3.4 × 10^7^, and 5.2 × 10^8^ per gram of stem tissue at 21 d postinoculation, respectively (Fig. 7d). These results suggest that anthranilic acid plays a vital role in the pathogenesis of *R. solanacearum*.

### Anthranilic acid controls the expression levels of a wide range of genes

**Fig. 8:**
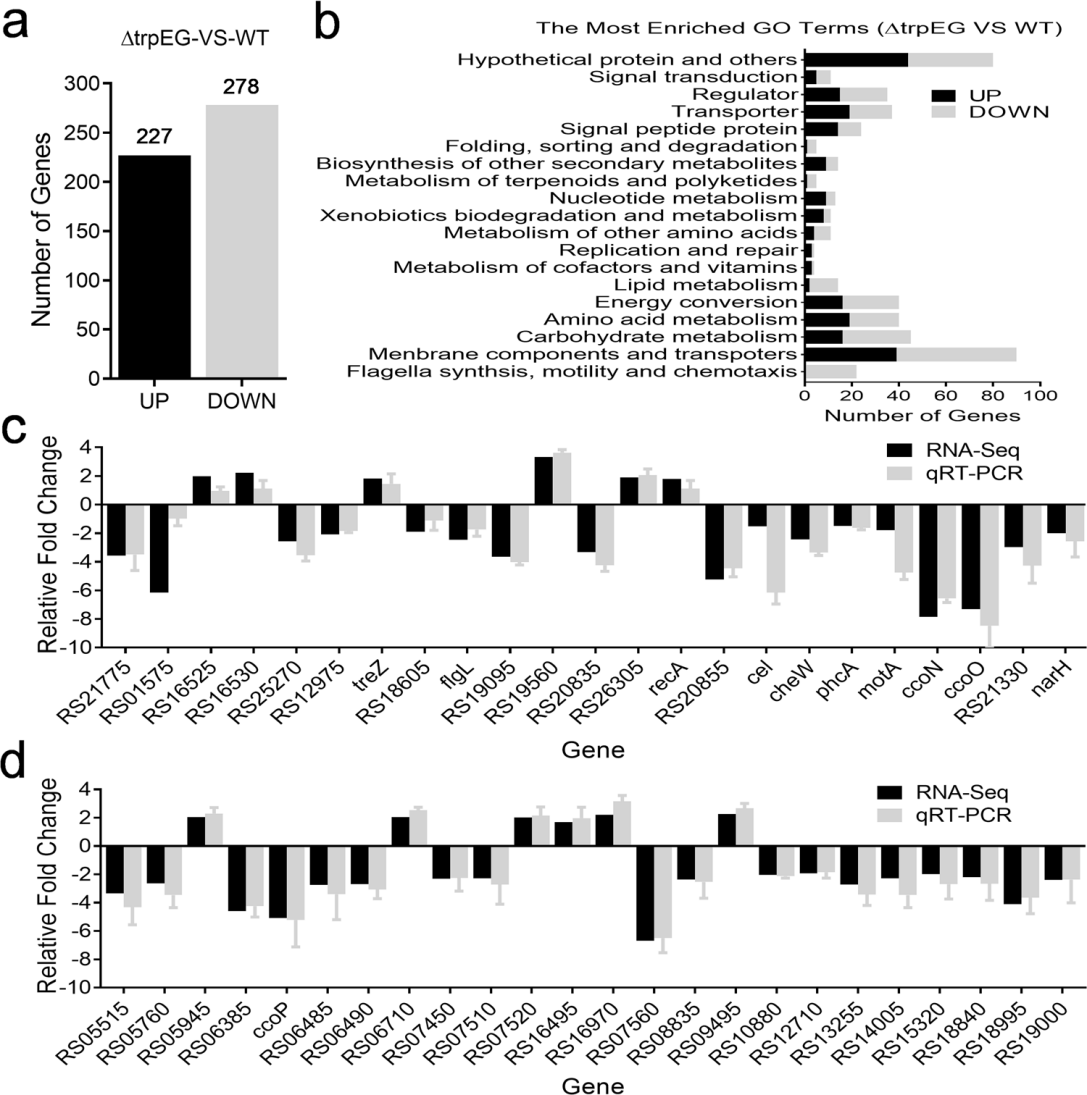
Differential gene expression profiles between the *R. solanacearum* GMI1000 *trpEG* mutant strain and the wild-type strain as measured by RNA-Seq (Log_2_ fold-change ≥1.5). **a** A number of genes were upregulated and downregulated in ∆trpEG compared with the wild-type strain. **b** GO term enrichment analysis of differentially expressed genes between the ∆trpEG and wild-type strains. Divergently regulated genes are not depicted in these diagrams but can be found in Supplementary Table 4. **c,** **d** qRT-PCR analysis of the genes that showed differential expression in the *trpEG* mutant strain compared with the wild-type strain.

To determine the comprehensive regulatory roles of *trpEG* in controlling bacterial physiology, we analysed and compared the transcriptomes of the wild-type strain and the ΔtrpEG mutant strain by using RNA Sequencing (RNA-Seq). Differential gene expression analysis showed that 227 genes were increased and 278 genes were decreased (Log_2_ fold-change ≥1.5) in the ΔtrpEG mutant strain compared with the wild-type GMI1000 strain (Fig. 8a and Supplementary Table 4). These differentially expressed genes were associated with a range of biological functions, including motility and cell attachment, stress tolerance, virulence, regulation, transcriptional regulators, membrane components, transports, multidrug resistance, detoxification and signal transduction (Fig. 8b and Supplementary Table 4). Quantitative RT-PCR analysis of select genes confirmed the RNA-seq results (Fig. 8c, d). These genes include the *phc* and *sol* systems and the *eps* gene cluster (*epsA*, *epsB*, *epsC*, *epsD*, *epsE* and *epsF*). We also found that many flagellum genes, such as *motA*, *flgK*, *flgL*, *fliQ*, *fliP*, *fliD*, *fliS*, *fliG*, *fliH*, and *fliJ*, were downregulated in the ΔtrpEG mutant strain.

### Anthranilic acid is produced by many bacteria

To investigate whether anthranilic acid is widely present in bacteria, *trpEG* homologues were searched in the genome database by using the BLAST program. Many bacteria exhibited homologues of anthranilic acid synthase, which shared high similarity of their amino acid sequences (Supplementary Fig. 18 and Table 5). We then selected several bacterial strains and cultured them in liquid medium, then collected the supernatants after the cells had grown for 48 h (OD_600_ of ~3.0). The ethyl acetate extracts of these strains were analysed by using LC-MS. The results showed that all the tested bacterial species (*Burkholderia cenocepacia*, *Collimonas pratensis*, *P. aeruginosa*, *Achromobacter xylosoxidans*, and *Comamonas aquatic*) produced high levels of anthranilic acid (Supplementary Fig. 19), indicating that the anthranilic acid compound might be widely conserved in bacteria.

## Discussion

In soil and rhizosphere environments, *R. solanacearum* usually coexists with many other microorganisms. These bacteria and fungi compete for the same ecological niche and nutrient resources [23]. Therefore, the ability to cope with a range of competitors is essential for growth and survival in soil ecosystems. The results of this study identified a new weapon *R. solanacearum* GMI1000 against one of its competitors, the fungus *S. scitamineum* (Fig. 1). It was revealed that the *R. solanacearum* GMI1000 strain utilizes anthranilic acid to inhibit the sexual mating and morphological transition of *S. scitamineum* to achieve a competitive advantage (Figs. 1, 3). Some previous studies have also revealed that *R. solanacearum* employs QS to control the production of ralsolamycin to achieve a fitness advantage in inter-kingdom competition [24–26]. Our new findings suggested that complicated mechanisms have evolved in *R. solanacearum* to achieve for a competitive advantage in the microbial community.

In addition to its vital role in microbial ecology, anthranilic acid is very important for the bacterial physiology of *R. solanacearum*. It was indicated that the deletion of TrpEG caused a remarkable reduction in anthranilic acid production and serious impairment of the biological functions and pathogenicity of *R. solanacearum*, while *in trans* expression of *trpEG* or the addition of anthranilic acid restored these phenotypes in the *trpEG* deletion mutant strain (Figs. 4, 6, 7, 8). Increasing evidence has accumulated to support the multiple roles of QS signals or cues in both intraspecies signalling and interspecies interactions. Our previous studies have also indicated that in addition to their key roles in intraspecies signalling, DSF-family signals exhibit an important function in interspecies and inter-kingdom communication [48–51]. Our findings in this study establish the significant roles of the anthranilic acid compound in inter-kingdom communication as well as in intraspecies signalling regulation for the first time.

Both in vivo and in vitro experiments have demonstrated the enzymatic activity of TrpEG from *R. solanacearum* GMI1000 to synthesize anthranilic acid (Fig. 4). The comparison of peptide sequences revealed that TrpEG from *R. solanacearum* GMI1000 shares high identity (57.12%) with the anthranilate synthase TrpEG from *P. aeruginosa* PAO1 [11, 12, 47]. In addition, we identified the homologues of *kynA*, *kynB*, and *kynU* in *R. solanacearum* GMI1000, which shared identities of 66.32%, 69.63% and 69.78% with their homologues in *P. aeruginosa* PAO1, respectively [44]. However, our findings from this study suggest that the anthranilic acid/TrpEG system can be distinguished from the relevant system in *P. aeruginosa*. This conclusion was supported by several lines of evidence. First, anthranilic acid is a precursor for the biosynthesis of DHQ, HHQ and PQS in *P. aeruginosa*, while these molecules were not found in *R. solanacearum* GMI1000 (Supplementary Fig. 13–15). Second, no homologues of *pqs* gene cluster, which are the synthases for DHQ, HHQ and PQS, respectively, in *P. aeruginosa* were identified in *R. solanacearum* GMI1000. Finally, the addition of exogenous PQS, HHQ, or DHQ resulted in no restoration of the phenotypes of the *R. solanacearum trpEG* mutant strain (Supplementary Fig. 16). In general, our results demonstrate that anthranilic acid/TrpEG system in *R. solanacearum* has evolved independently and is distinguished from the *pqs* or other QS systems in *P. aeruginosa*.

BLAST searches revealed that the *trpEG* homologues are conserved in all other *Ralstonia* genomovars with high identities of more than 85%. Furthermore, our findings indicate that both anthranilic acid and TrpEG are widely conserved in many other bacterial species, including *B. cenocepacia*, *C. pratensis*, *P. aeruginosa*, *A. xylosoxidans*, and *C. aquatic* (Supplementary Figs. 18, 19). In general, we have demonstrated that the anthranilic acid compound plays a vital role in the control of QS signal production, biological functions and virulence in *R. solanacearum*. Further investigation of this compound will expand our knowledge of the regulatory mechanism of pathogenicity in bacterial pathogens.

## Supplementary information

Supplementary Materials and Methods

Supplementary Figure legends

Supplementary Figure 1

Supplementary Figure 2

Supplementary Figure 3

Supplementary Figure 4

Supplementary Figure 5

Supplementary Figure 6

Supplementary Figure 7

Supplementary Figure 8

Supplementary Figure 9

Supplementary Figure 10

Supplementary Figure 11

Supplementary Figure 12

Supplementary Figure 13

Supplementary Figure 14

Supplementary Figure 15

Supplementary Figure 16

Supplementary Figure 17

Supplementary Figure 18

Supplementary Figure 19

Supplementary Table legends

Supplementary Table 1

Supplementary Table 2

Supplementary Table 3

Supplementary Table 4

Supplementary Table 5
