# Supplementary material for: Anthranilic acid from Ralstonia solanacearum plays dual roles in intraspecies signalling and inter-kingdom communication
Source: ISME J. 2020 May 26;14(9):2248–60. doi: 10.1038/s41396-020-0682-7 (PMC7608240; doi:10.1038/s41396-020-0682-7)
Supplement: Supplementary file 1 — Supplementary Materials and Methods [file 41396_2020_682_MOESM1_ESM.docx]

**Supplementary Materials and Methods**

**LC-MS Analysis**

*R. solanacearum* cells were cultured in TTC medium for 48 h, and the supernatant was extracted with an equal volume of ethyl acetate. The culture supernatants were extracted twice with ethyl acetate, the solvent was evaporated, and the residue was dissolved in methanol. Analyses were performed by using a Waters ACQUITY UPLC/Xevo G2 QTOF system (Waters, Milford- Massachusetts, USA), which consisted of an ACQUITY UPLC system and a Waters Q-Tof Premier high-resolution mass spectrometer in negative electrospray ionization mode interfaced with an ACQUITY UPLC BEH C18 column (2.1×50 mm). Elution was performed via a gradient of 5-100% CH_3_OH in water supplemented with 0.01% formic acid at a flow rate of 0.4 ml/min for 10 min. Then, 100% CH_3_OH was used for 2 min, and 5% CH_3_OH was used for 3 min. The entire column eluate was introduced to the Q-Tof mass spectrometer according to the manufacturer’s instructions [1]. The experiment was performed according to the previous method. The anthranilic acid levels in the culture supernatant were measured using the peak area in the extracted ion chromatogram.

**GC-MS Analysis**

*R. solanacearum* cells were cultured in TTC medium for 48 h to an OD_600_ of 3.0, and the supernatant was extracted with an equal volume of ethyl acetate. The culture supernatants were extracted twice with ethyl acetate, the solvent was evaporated, and the residue was dissolved in hexane. Combined gas chromatography-mass spectrometry (GC-MS) analyses were performed on an Agilent Technologies 7890B-5977B GC/MSD system (Agilent, California, USA) equipped with a DB-5 ms column (30 m×0.25 mm×0.25 μm), operating in electron impact electron ionization positive (EI^+^) mode at 70 eV; helium (99.999%) was used as carrier gas at a constant flow of 1 ml/min; an injection volume of 0.5 EI was employed (split ratio of 10:1); the injector temperature was 280°C; and the transfer line temperature was 300°C. The oven temperature was programmed to gradually increase from 50°C (isothermal for 2 min) in steps of 10°C/min to 300°C. Mass spectra were obtained at 70 eV, a scan interval of 0.5 s, and a full mass scan range of 25 m/z to 1000 m/z. Data acquisition was performed with GC/MS Acquisition B.07.00+MSD Chemstation Data Analysis 1701FA F.01.00 (Agilent, California, USA) [2].

**Protein expression and purification**

The coding region of *trpEG* was amplified with the primers listed in Supplementary Table 2 and fused to the expression vector pET-28a. The fusion gene constructs were transformed into *E. coli* strain BL21. Affinity purification of the HIS-TrpEG fusion protein was performed following the method described previously [3]. The cleaved fusion proteins were eluted and analysed by SDS-PAGE. Fusion protein cleavage with PreScission Protease (GE Healthcare; 2 units/100 μl of bound protein) was conducted at 4°C overnight. The enzymatic reactions were tested at 28°C in 0.05 M Tris-HCl (adjusted to pH 7.5 at 28°C), 5% glycerol, 5 mM MgCl_2_, 5 mM Dithiothreitol (DTT), 3.0 μM chorismic acid (CA) and 20 μM glutamine [4].

**Phenotype assays**

For the analysis of biofilm formation [5], a single colony of each strain was inoculated and grown overnight at 28°C with agitation in 5 ml of TTC medium. Bacterial cells were inoculated 1:100 in TTC media in 96-well polystyrene plates. After incubation at 28°C for 24 h, the cells were stained with 0.1% crystal violet (CV) for 30 min. The planktonic cells were removed by several rinses with H_2_O. The CV-stained bound cells were air dried for 1 h and then dissolved in 95% ethanol, and the optical density 570 (OD_570_) of the solution was measured to quantify biofilm formation.

Cellulase activity was determined on carboxymethylcellulose sodium (CMS) solid medium (1 litre contains 1 g carboxymethylcellulose sodium, 3.8 g Na_3_PO_4_, and 8.0 g agar, PH 7.0) [6]. The CMS medium was added to a culture dish (Φ 9 cm). The overnight culture was diluted to an OD_600_ of approximately 0.1, and 2 μL of a bacterial suspension was inoculated into the centre of the CMS plates. The plates were incubated at 28°C for 48 h. The plates were stained with 0.5% Congo red for 30 min. The plates were rinsed three times with 1 M NaCl. Then, the size of the transparent ring was measured.

Swarming motility was determined on semi-solid agar (0.3%) [7]. Bacteria were inoculated into the centre of plates containing 1% tryptone (Becton, Dickinson and Company, Maryland, USA) and 0.3% agar (Becton, Dickinson and Company, Maryland, USA). The plates were incubated at 28°C for 48 h before the diameter of the colonies was measured.

For the quantification of extracellular polysaccharide (EPS) production, bacteria were inoculated into sucrose and peptone (SP) liquid medium (1 litre contains 5 g peptone, 20 g sucrose, 0.5 g KH_2_PO_4_, and 0.25 g MgSO_4_, pH 7.2) [8]. A 100 ml aliquot of the culture (OD_600_=3.0) was collected and centrifuged at 12,000 rpm for 20 min. The supernatants were filtered through a 0.22 μM membrane. The collected supernatants were mixed with 4 volumes of absolute ethanol, and the mixture was incubated at 4°C overnight. The precipitated EPS was isolated by centrifugation and dried overnight at 55°C before the determination of dry weight.

**Bacterial growth analysis**

An overnight bacterial culture in TTC medium was washed twice in fresh TTC medium or MP minimal medium and inoculated into fresh mediums to an OD_600_ of 0.01 in TTC medium and 0.1 in MP minimal medium. A 200-μL cell suspension was grown in each well at 28°C in a low intensity shaking model using the Bioscreen-C automated growth curve analysis system. TTC medium or MP minimal medium were used as the negative control. MP minimal medium (1 liter): FeSO_4_·7H_2_O, 1.25×10^-4^ g; (NH_4_)_2_SO_4_, 0.5g; MgSO_4_·7H_2_O, 0.05g; KH_2_PO_4_, 3.4g; The pH was adjusted to 7, and 20 mM glutamate was added [9,10].

**Construction of *epsA* reporter strains and measurement of β-galactosidase activity**

The promoter of *epsA* was amplified using the primer pairs listed in Supplementary Table 2 with XhoI restriction sites attached. The resulting products were digested with XhoI, and ligated at the same enzyme sites in the vector pME2-*lacZ*. Transconjugants were then selected on TTC agar plates supplemented with tetracycline and X-gal. Bacterial cells were grown at 28°C in the presence and absence of different concentrations of anthranilic acid to an OD_600_=1.0, and measurement of β-galactosidase activities was performed following the methods as described previously [11].

**RNA-seq analysis**

Double-stranded cDNA synthesis and high-throughput RNA-seq were performed as described previously [12]. For each strain, three biological replicates were sequenced. Trimmed sequence reads were aligned to the *R. solanacearum* GMI1000 genome sequence using Bowtie2-2.2.3 [13] and normalized read counts were compared using HTSeq v0.6.1 as described previously [14]. For each replicate sample, between 7.3 million and 10.2 million sequence reads were uniquely mapped to the *R. solanacearum* GMI1000 genome sequence. Differentially expressed genes were identified as those with a high expression level (log_2_≥1.5) across all replicates at a false discovery rate (FDR) of <0.01.

**Quantitative real-time PCR assays**

*R.* *solanacearum* cells were cultured to an OD_600_=1.0 and then harvested. RNA was isolated using the Eastep Super Total RNA Extraction Kit (Promega, Madison, USA). cDNA synthesis and quantitative RT-PCR analysis were performed with the ChamQTM Universal SYBR qPCR Master Mix (Vazyme, Nanjing, China) according to the manufacturer’s instructions in a 7300Plus Quantitative Real-Time PCR System. As a control, quantitative RT-PCR was similarly applied to analyse the expression of the 16S rRNA gene. The relative expression levels of the target genes were calculated using the quantitative comparative CT (ΔΔCT) method [1].

TRIzol reagent (ThermoFisher, Carlsbad CA, USA) was used for total RNA extraction from *S. scitamineum* MAT-1 and MAT-2. MAT-1 and MAT-2 haploids were mixed and plated on YePS agar medium supplemented with anthranilic acid at 0 μM or 100 μM for 24 h before total RNA extraction with TRIzol reagent [15]. cDNA synthesis and quantitative RT-PCR analysis were carried out using the ChamQTM Universal SYBR qPCR Master Mix (Vazyme, Nanjing, China) according to the manufacturer’s instructions. Using a 7300Plus Quantitative Real-Time PCR System. As a control, quantitative RT-PCR was similarly applied to analyse the expression of the actin gene. The relative expression levels of the target genes were calculated using the quantitative comparative CT (ΔΔCT) method.

**Supplementary References**

1. Song S, Fu S, Sun X, Li P, Wu J, Dong T, et al. Identification of cyclic dipeptides from *Escherichia coli* as new antimicrobial agents against *Ralstonia solanacearum*. *Molecules*. 2018;23:214.
2. Flavier AB, Clough SJ, Schell MA, Denny TP. Identification of 3-hydroxypalmitic acid methyl ester as a novel autoregulator controlling virulence in *Ralstonia solanacearum*. *Mol Microbiol*. 1997;26:251-259.
3. Yang C, Cui C, Ye Q, Kan J, Fu S, Song S, et al. *Burkholderia cenocepacia* integrates cis-2-dodecenoic acid and cyclic dimeric guanosine monophosphate signals to control virulence. *Proc Natl Acad Sci USA*. 2017;114:13006-13011.
4. Knöchel T, Ivens A, Hester G, Gonzalez A, Bauerle R, Wilmanns M, et al. The crystal structure of anthranilate synthase from *Sulfolobus solfataricus*: functional implications. *Proc Natl Acad Sci USA*. 1999;96:9479-9484.
5. Yao J, Allen C. The plant pathogen *Ralstonia solanacearum* needs aerotaxis for normal biofilm formation and interactions with its tomato host. *J Bacteriol*. 2007;189:6415-6424.
6. Achari GA, Ramesh R. Characterization of bacteria degrading 3-hydroxy palmitic acid methyl ester (3OH-PAME), a quorum sensing molecule of *Ralstonia solanacearum*. *Lett Appl Microbiol*. 2015;60:447-455.
7. Kelman A, Hruschka J. The role of motility and aerotaxis in the selective increase of avirulent bacteria in still broth cultures of *Pseudomonas solanacearum*. *J Gen Microbiol*. 1973;76:177-188.
8. Zhu YJ, Xiao RF, Liu B. Growth and pathogenicity characteristics of *Ralstonia solanacearum* strain RS1100 in long-term stationary phase culture. *J Plant Dis Protect*. 2010;117:156-161.
9. Plener L, Boistard P, Gonzalez A, Boucher C, Genin S. Metabolic adaptation of *Ralstonia solanacearum* during plant infection: a methionine biosynthesis case study. *PLoS One*. 2012;7:e36877.
10. Plener L, Manfredi P, Valls M, Genin S. PrhG, a transcriptional regulator responding to growth conditions, is involved in the control of the type III secretion system regulon in *Ralstonia solanacearum*. *J Bacteriol.* 2010;192:1011-1019.
11. Cui C, Song S, Yang C, Sun X, Huang Y, Li K, et al. Disruption of quorum sensing and virulence in *Burkholderia cenocepacia* by a structural analogue of the cis-2-dodecenoic acid signal. *Appl Environ Microbiol.* 2019;85:e00105-19.
12. Cui C, Yang C, Song S, Fu S, Sun X, Deng Y. A novel two-component system modulates quorum sensing and pathogenicity in *Burkholderia cenocepacia*. *Mol Microbiol*. 2018;108:32-44.
13. Langmead B, Salzberg SL. Fast gapped-read alignment with Bowtie 2. *Nat Methods*. 2012;9:357-359.
14. Trapnell C, Williams BA, Pertea G, Mortazavi A, Kwan G, Van Baren MJ, et al. Transcript assembly and quantification by RNA-Seq reveals unannotated transcripts and isoform switching during cell differentiation. *Nat Biotechnol*. 2010;28:511-515.
15. Yan M, Dai W, Cai E, Ding Y, Jiang Z, Zhang LH. Transcriptome analysis of *Sporisorium scitamineum* reveals critical environmental signals for fungal sexual mating and filamentous growth. *BMC Genomics*. 2016;17:1-11
