# Supplementary material for: Anthranilic acid from Ralstonia solanacearum plays dual roles in intraspecies signalling and inter-kingdom communication
Source: ISME J. 2020 May 26;14(9):2248–60. doi: 10.1038/s41396-020-0682-7 (PMC7608240; doi:10.1038/s41396-020-0682-7)
Supplement: Supplementary file 2 — Supplementary Figure legends [file 41396_2020_682_MOESM2_ESM.docx]

**Supplementary Figure legends**

**Supplementary Figure 1** The Distortionless Enhancement by Polarization Transfer 135 spectra signal of anthranilic acid isolated from the ethyl acetate extract of *R. solanacearum*.

**Supplementary Figure 2** Analysis of the growth curve of *S. scitamineum* cells to which anthranilic acid was added at different concentrations. The experiment was started at an initial OD_600_ of 0.01. The cells were inoculated in YePS medium at 28°C with three replicates in a low intensity shaking model using the Bioscreen-C automated growth curve analysis system. The data are means ± standard deviations of three independent experiments.

**Supplementary Figure 3** Effect of different concentrations of benzoic acid (a) and P-aminobenzoic acid (b) on *S. scitamineum* sexual mating and morphological transition. The sexual mating and morphological transition of *S. scitamineum* was analyzed when it was grown in a plate. Anthranilic acid was used as a positive control.

**Supplementary Figure 4** Effect of *trpEG* on the growth curve of *R. solanacearum* GMI1000 in TTC medium (a), and MP minimal medium (b). The cells were inoculated at 28°C with three replicates in a low intensity shaking model using the Bioscreen-C automated growth curve analysis system. The experiment was started at an initial OD_600_ of 0.01 in TTC medium and 0.1 in MP minimal medium. The data are means ± standard deviations of three independent experiments.

**Supplementary Figure 5** Effect of *trpEG* and *kynAUB* on anthranilic acid production in *R. solanacearum* GMI1000. The data are means ± standard deviations of three independent experiments. ***p < 0.001 (unpaired t-test).

**Supplementary Figure 6** The precursor ion mass spectrogram of chorismic acid (255.0658) in Negative ion mode.

**Supplementary Figure 7** Calibration curves were made by plotting peak area (Y) versus the concentrations (X, μM) of the standard solutions of anthranilic acid. The regression equations of anthranilic acid was *Y*=433.14*X*+6432.4, and the linear *R2*=0.9980 (*n*=7).

**Supplementary Figure 8** Effect of *trpE* and the anthranilic acid on the QS-regulated phenotypes of motility (a), biofilm formation (b), EPS production (c) and cellulase production (d) in *R. solanacearum* GMI1000. The data are means ± standard deviations of three independent experiments. *p < 0.05; **p < 0.01; ***p < 0.001 (unpaired t-test).

**Supplementary Figure 9** Effect of *trpG* and the anthranilic acid on the QS-regulated phenotypes of motility (a), biofilm formation (b), EPS production (c) and cellulase production (d) in *R. solanacearum* GMI1000. The data are means ± standard deviations of three independent experiments. *p < 0.05; **p < 0.01; ***p < 0.001 (unpaired t-test).

**Supplementary Figure 10** Effect of the *R. solanacearum* wild-type, *trpEG* mutant, *trpE* mutant, *trpG* mutant, and complement strains on the sexual mating and morphological transition of *S. scitamineum*.

**Supplementary Figure 11** Influence of *trpEG* and *kynAUB* on the QS-regulated phenotypes of motility (a), biofilm formation (b), EPS production (c) and cellulase production (d) in *R. solanacearum* GMI1000. The data are means ± standard deviations of three independent experiments. ***p < 0.001 (unpaired t-test).

**Supplementary Figure 12** Effect of *trpEG* on QS gene expression levels (a) and QS signal production (b) in *R. solanacearum* GMI1000. RAA: *Ralstonia* anthranilic acid. The data are means ± standard deviations of three independent experiments. **p < 0.01; ***p < 0.001 (unpaired t-test).

**Supplementary Figure 13** Analysis of PQS by LC-MASS. (a) HPLC chromatograms of the standard PQS. (b) ESI-MS spectra of the standard PQS. (c) HPLC chromatograms of the ethyl acetate extract of *R. solanacearum* GMI1000. (d) ESI-MS spectra of the ethyl acetate extract of *R. solanacearum* GMI1000.

**Supplementary Figure 14** Analysis of HHQ by LC-MASS. (a) HPLC chromatograms of the standard HHQ. (b) ESI-MS spectra of the standard HHQ. (c) HPLC chromatograms of the ethyl acetate extract of *R. solanacearum* GMI1000. (d) ESI-MS spectra of the ethyl acetate extract of *R. solanacearum* GMI1000.

**Supplementary Figure 15** Analysis of DHQ by LC-MASS. (a) HPLC chromatograms of the standard DHQ. (b) ESI-MS spectra of the standard DHQ. (c) HPLC chromatograms of the ethyl acetate extract of *R. solanacearum* GMI1000. (d) ESI-MS spectra of the ethyl acetate extract of *R. solanacearum* GMI1000.

**Supplementary Figure 16** Effect of the addition of exogenous PQS, HHQ and DHQ on the phenotypes of motility (a), biofilm formation (b), EPS production (c) and cellulase production (d) in the ∆trpEG mutant strain. The data are means ± standard deviations of three independent experiments. ***p < 0.001 (unpaired t-test).

**Supplementary Figure 17** Effect of the addition of exogenous anthranilic acid on the expression level of *epsA* in the double deletion mutant strain ΔtrpEGΔkynAUB. The data are means ± standard deviations of three independent experiments. *p < 0.05; ***p < 0.001 (unpaired t-test).

**Supplementary Figure 18** Amino acid sequence alignment of TrpEG homologues of various bacterial species. Dark and blue coloration was used to label conserved sequences.

**Supplementary Figure 19** Analysis of anthranilic acid production in various bacteria by LC-MASS. (a) HPLC chromatograms of anthranilic acid in different bacteria. (b) ESI-MS spectra of anthranilic acid in different bacteria.
