# Supplementary material for: Anthranilic acid from Ralstonia solanacearum plays dual roles in intraspecies signalling and inter-kingdom communication
Source: ISME J. 2020 May 26;14(9):2248–60. doi: 10.1038/s41396-020-0682-7 (PMC7608240; doi:10.1038/s41396-020-0682-7)

**Supplementary Figure 1** The Distortionless Enhancement by Polarization Transfer 135 spectra signal of anthranilic acid isolated from the ethyl acetate extract of *R. solanacearum*.


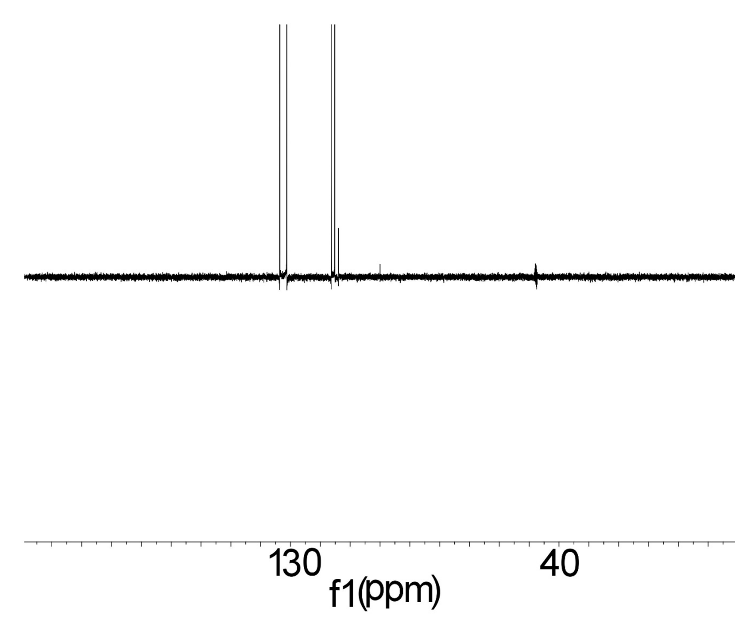

Supplement: Supplementary file 3 — Supplementary Figure 1 [file 41396_2020_682_MOESM3_ESM.docx]
