# Supplementary material for: Anthranilic acid from Ralstonia solanacearum plays dual roles in intraspecies signalling and inter-kingdom communication
Source: ISME J. 2020 May 26;14(9):2248–60. doi: 10.1038/s41396-020-0682-7 (PMC7608240; doi:10.1038/s41396-020-0682-7)

**Supplementary Figure 2** Analysis of the growth curve of *S. scitamineum* cells to which anthranilic acid was added at different concentrations. The experiment was started at an initial OD_600_ of 0.01. The cells were inoculated in YePS medium at 28°C with three replicates in a low intensity shaking model using the Bioscreen-C automated growth curve analysis system. The data are means ± standard deviations of three independent experiments.


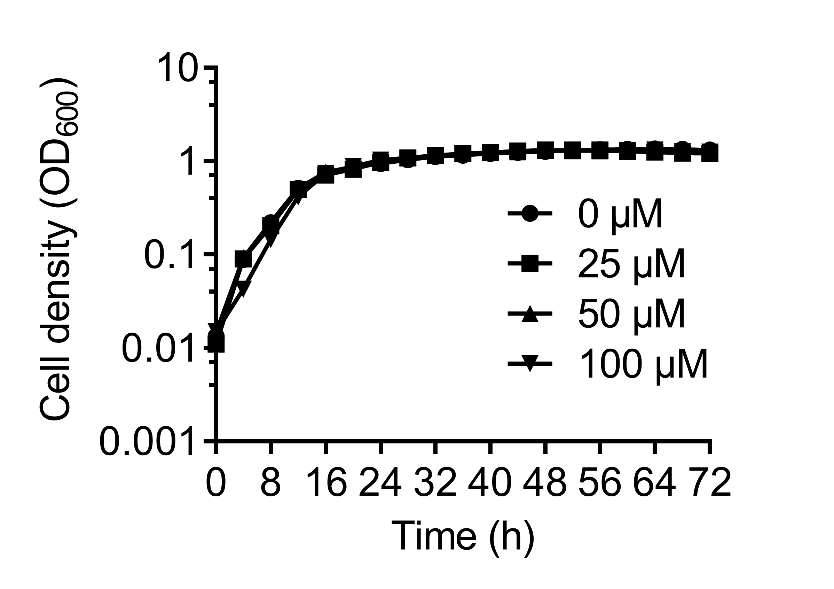

Supplement: Supplementary file 4 — Supplementary Figure 2 [file 41396_2020_682_MOESM4_ESM.docx]
