# Supplementary material for: Anthranilic acid from Ralstonia solanacearum plays dual roles in intraspecies signalling and inter-kingdom communication
Source: ISME J. 2020 May 26;14(9):2248–60. doi: 10.1038/s41396-020-0682-7 (PMC7608240; doi:10.1038/s41396-020-0682-7)

**Supplementary Figure 3** Effect of different concentrations of benzoic acid (a) and P-aminobenzoic acid (b) on *S. scitamineum* sexual mating and morphological transition. The sexual mating and morphological transition of *S. scitamineum* was analyzed when it was grown in a plate. Anthranilic acid was used as a positive control.


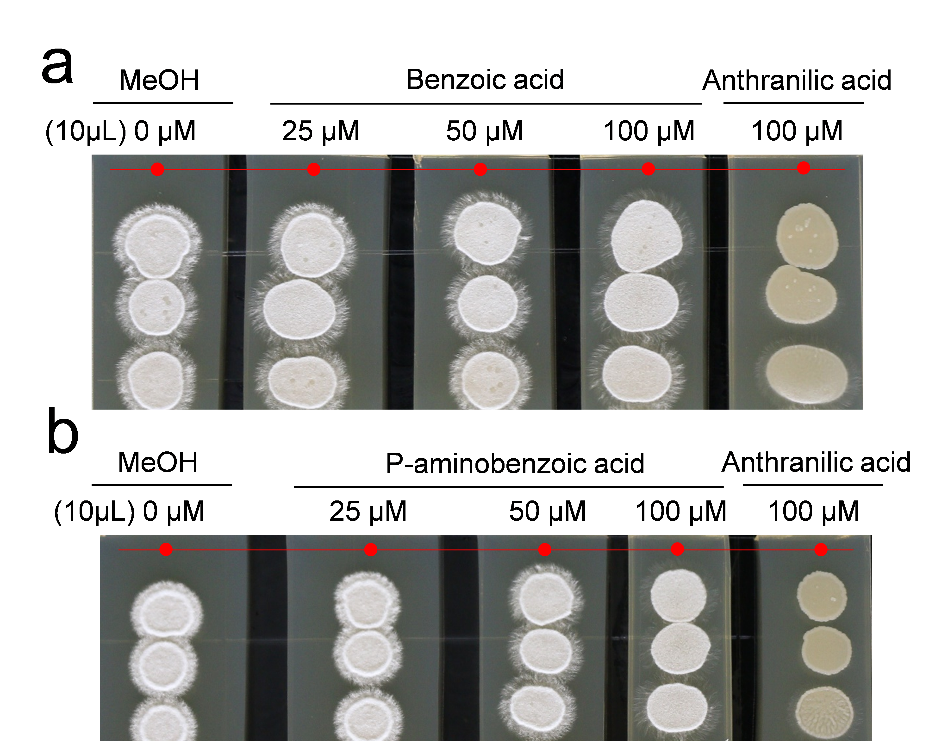

Supplement: Supplementary file 5 — Supplementary Figure 3 [file 41396_2020_682_MOESM5_ESM.docx]
