# Supplementary material for: Anthranilic acid from Ralstonia solanacearum plays dual roles in intraspecies signalling and inter-kingdom communication
Source: ISME J. 2020 May 26;14(9):2248–60. doi: 10.1038/s41396-020-0682-7 (PMC7608240; doi:10.1038/s41396-020-0682-7)
Supplement: Supplementary file 6 — Supplementary Figure 4 [file 41396_2020_682_MOESM6_ESM.docx]

**Supplementary Figure 4** Effect of *trpEG* on the growth curve of *R. solanacearum* GMI1000 in TTC medium (a), and MP minimal medium (b). The cells were inoculated at 28°C with three replicates in a low intensity shaking model using the Bioscreen-C automated growth curve analysis system. The experiment was started at an initial OD_600_ of 0.01 in TTC medium and 0.1 in MP minimal medium. The data are means ± standard deviations of three independent experiments.

*
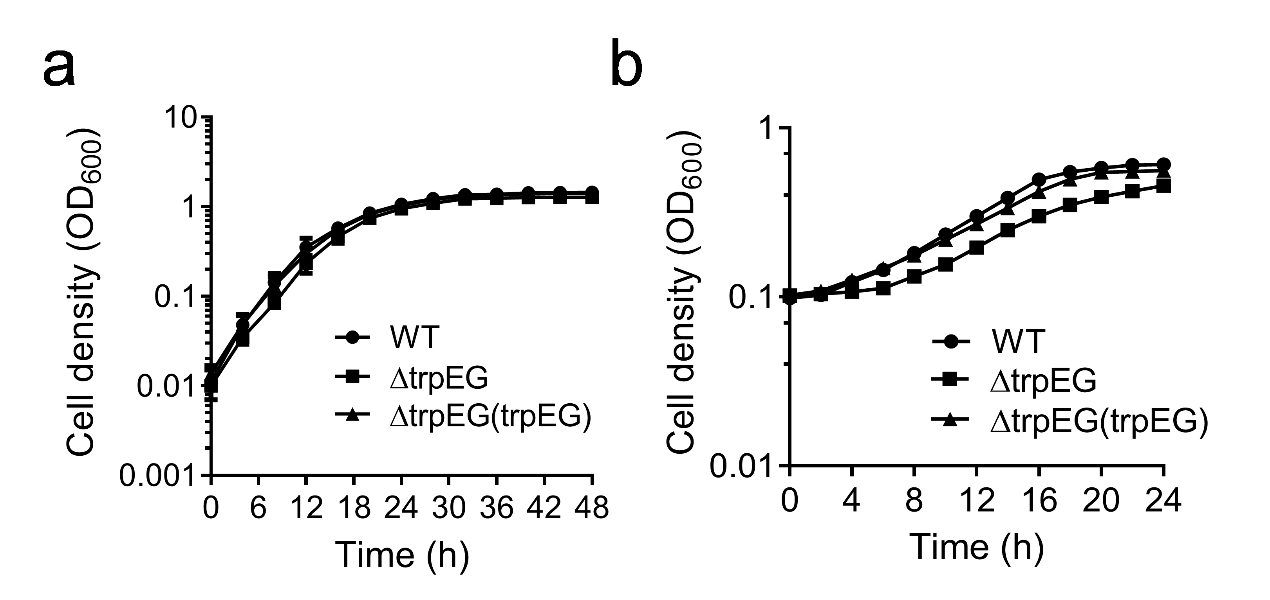
*
