# Supplementary material for: Anthranilic acid from Ralstonia solanacearum plays dual roles in intraspecies signalling and inter-kingdom communication
Source: ISME J. 2020 May 26;14(9):2248–60. doi: 10.1038/s41396-020-0682-7 (PMC7608240; doi:10.1038/s41396-020-0682-7)
Supplement: Supplementary file 8 — Supplementary Figure 6 [file 41396_2020_682_MOESM8_ESM.docx]

**Supplementary Figure 6** The precursor ion mass spectrogram of chorismic acid (255.0658) in Negative ion mode.

**
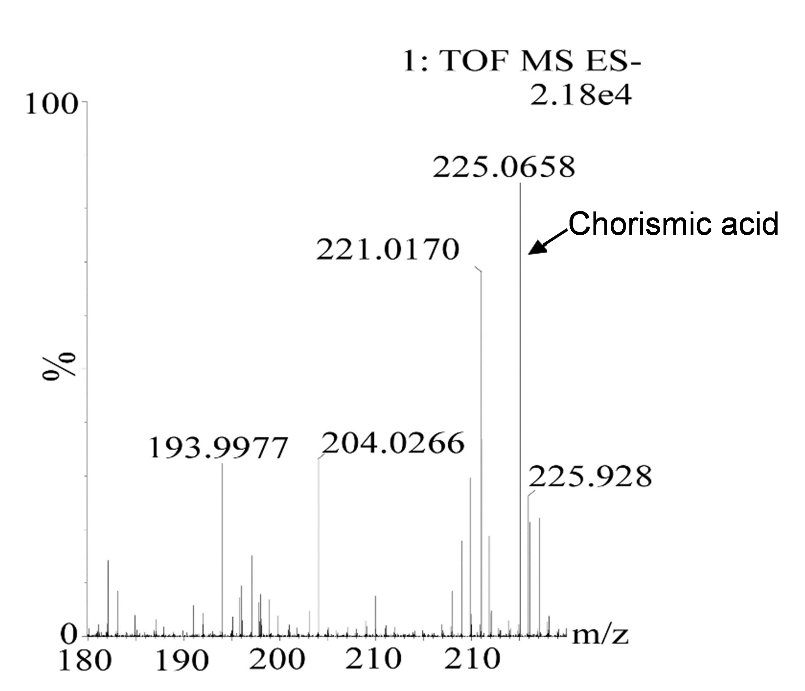
**
