# Supplementary material for: Anthranilic acid from Ralstonia solanacearum plays dual roles in intraspecies signalling and inter-kingdom communication
Source: ISME J. 2020 May 26;14(9):2248–60. doi: 10.1038/s41396-020-0682-7 (PMC7608240; doi:10.1038/s41396-020-0682-7)
Supplement: Supplementary file 9 — Supplementary Figure 7 [file 41396_2020_682_MOESM9_ESM.docx]

**Supplementary Figure 7** Calibration curves were made by plotting peak area (Y) versus the concentrations (X, μM) of the standard solutions of anthranilic acid. The regression equations of anthranilic acid was *Y*=433.14*X*+6432.4, and the linear *R2*=0.9980 (*n*=7).

*
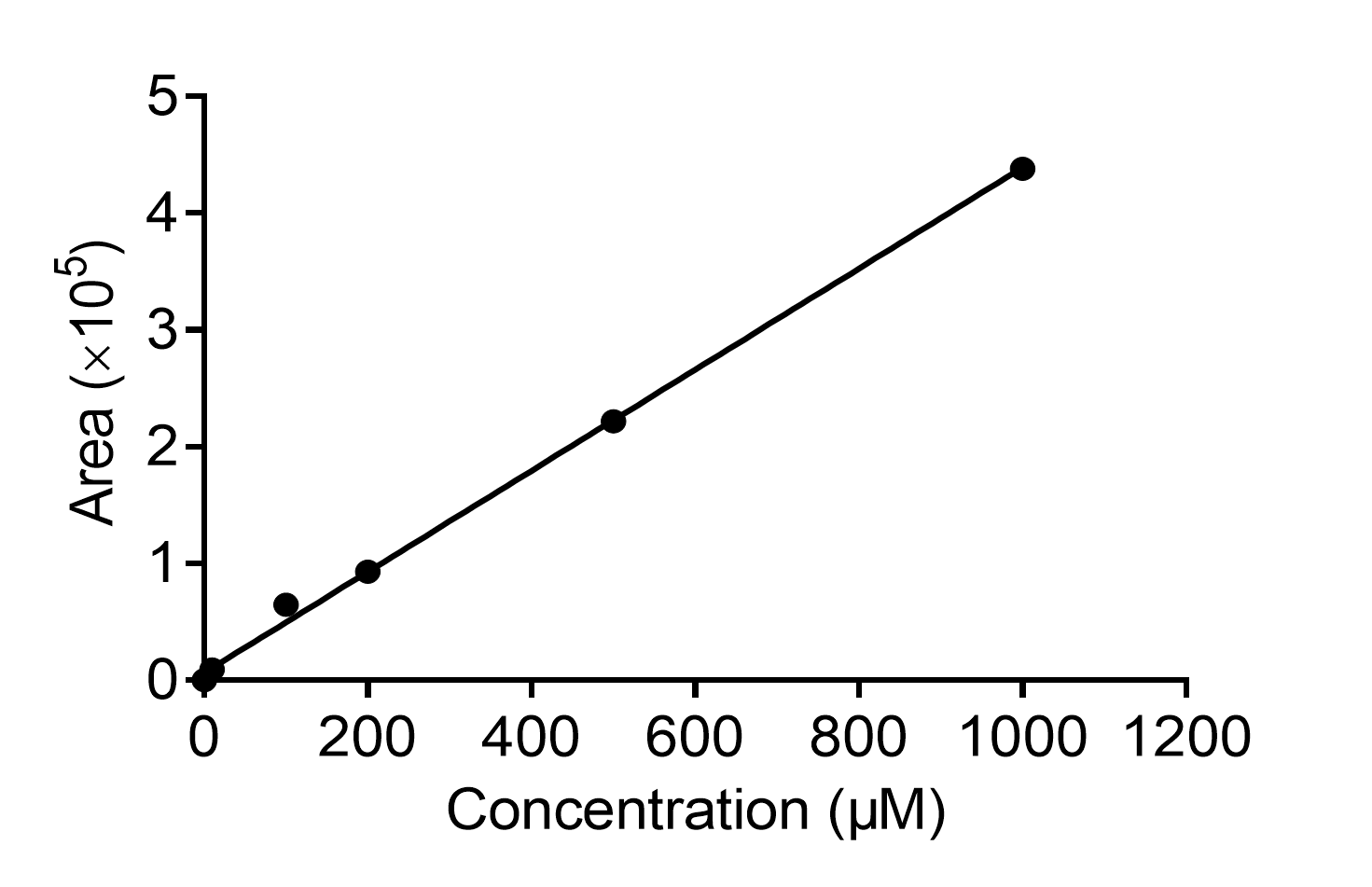
*
