# Supplementary material for: Anthranilic acid from Ralstonia solanacearum plays dual roles in intraspecies signalling and inter-kingdom communication
Source: ISME J. 2020 May 26;14(9):2248–60. doi: 10.1038/s41396-020-0682-7 (PMC7608240; doi:10.1038/s41396-020-0682-7)
Supplement: Supplementary file 10 — Supplementary Figure 8 [file 41396_2020_682_MOESM10_ESM.docx]

**Supplementary Figure 8** Effect of *trpE* and the anthranilic acid on the QS-regulated phenotypes of motility (a), biofilm formation (b), EPS production (c) and cellulase production (d) in *R. solanacearum* GMI1000. The data are means ± standard deviations of three independent experiments. *p < 0.05; **p < 0.01; ***p < 0.001 (unpaired t-test).

*
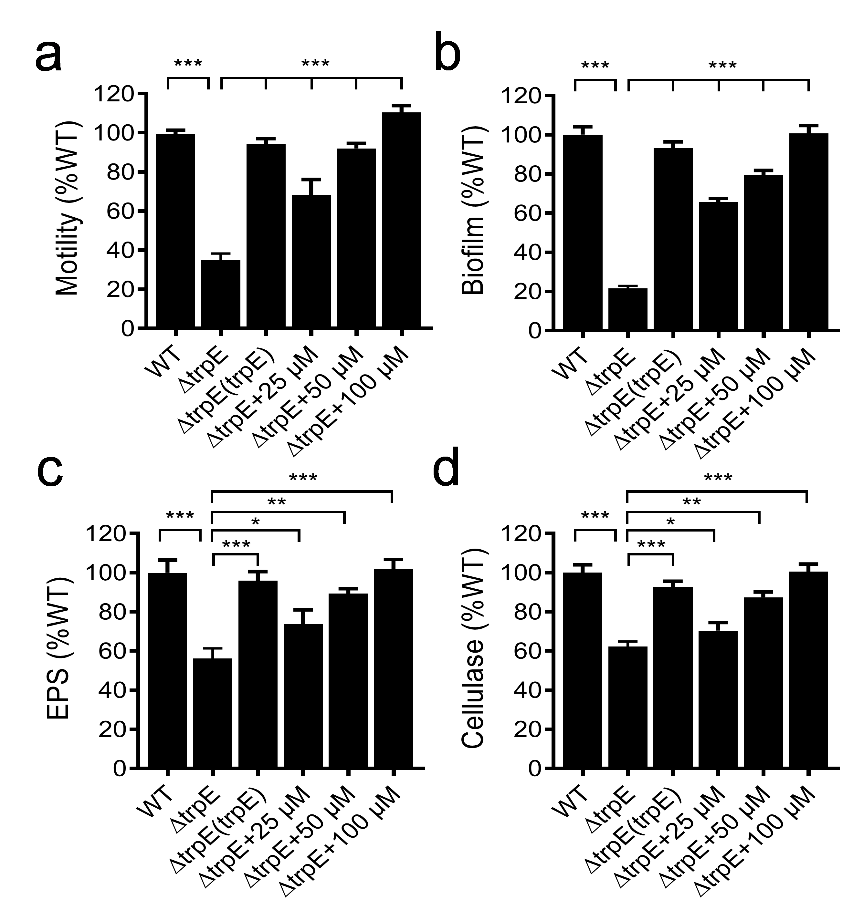
*
