# Supplementary material for: Anthranilic acid from Ralstonia solanacearum plays dual roles in intraspecies signalling and inter-kingdom communication
Source: ISME J. 2020 May 26;14(9):2248–60. doi: 10.1038/s41396-020-0682-7 (PMC7608240; doi:10.1038/s41396-020-0682-7)
Supplement: Supplementary file 12 — Supplementary Figure 10 [file 41396_2020_682_MOESM12_ESM.docx]

**Supplementary Figure 10** Effect of the *R. solanacearum* wild-type, *trpEG* mutant, *trpE* mutant, *trpG* mutant, and complement strains on the sexual mating and morphological transition of *S. scitamineum*.

*
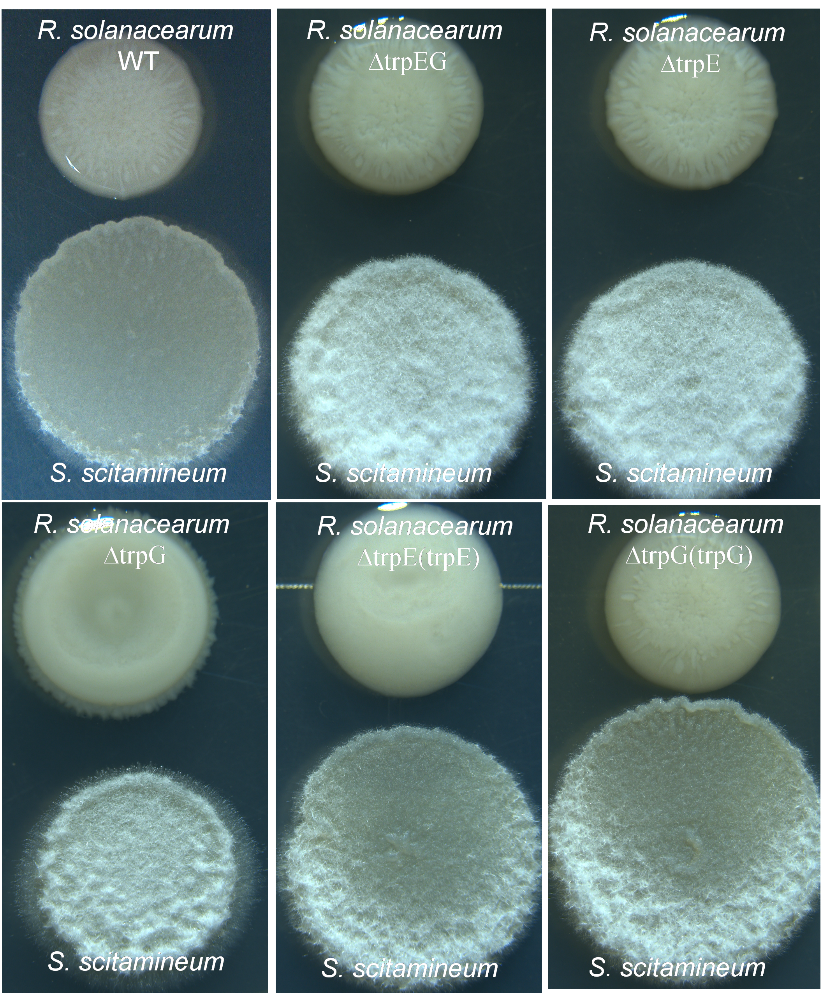
*
