# Supplementary material for: Anthranilic acid from Ralstonia solanacearum plays dual roles in intraspecies signalling and inter-kingdom communication
Source: ISME J. 2020 May 26;14(9):2248–60. doi: 10.1038/s41396-020-0682-7 (PMC7608240; doi:10.1038/s41396-020-0682-7)
Supplement: Supplementary file 14 — Supplementary Figure 12 [file 41396_2020_682_MOESM14_ESM.docx]

**Supplementary Figure 12** Effect of *trpEG* on QS gene expression levels (a) and QS signal production (b) in *R. solanacearum* GMI1000. RAA: *Ralstonia* anthranilic acid. The data are means ± standard deviations of three independent experiments. **p < 0.01; ***p < 0.001 (unpaired t-test).

*
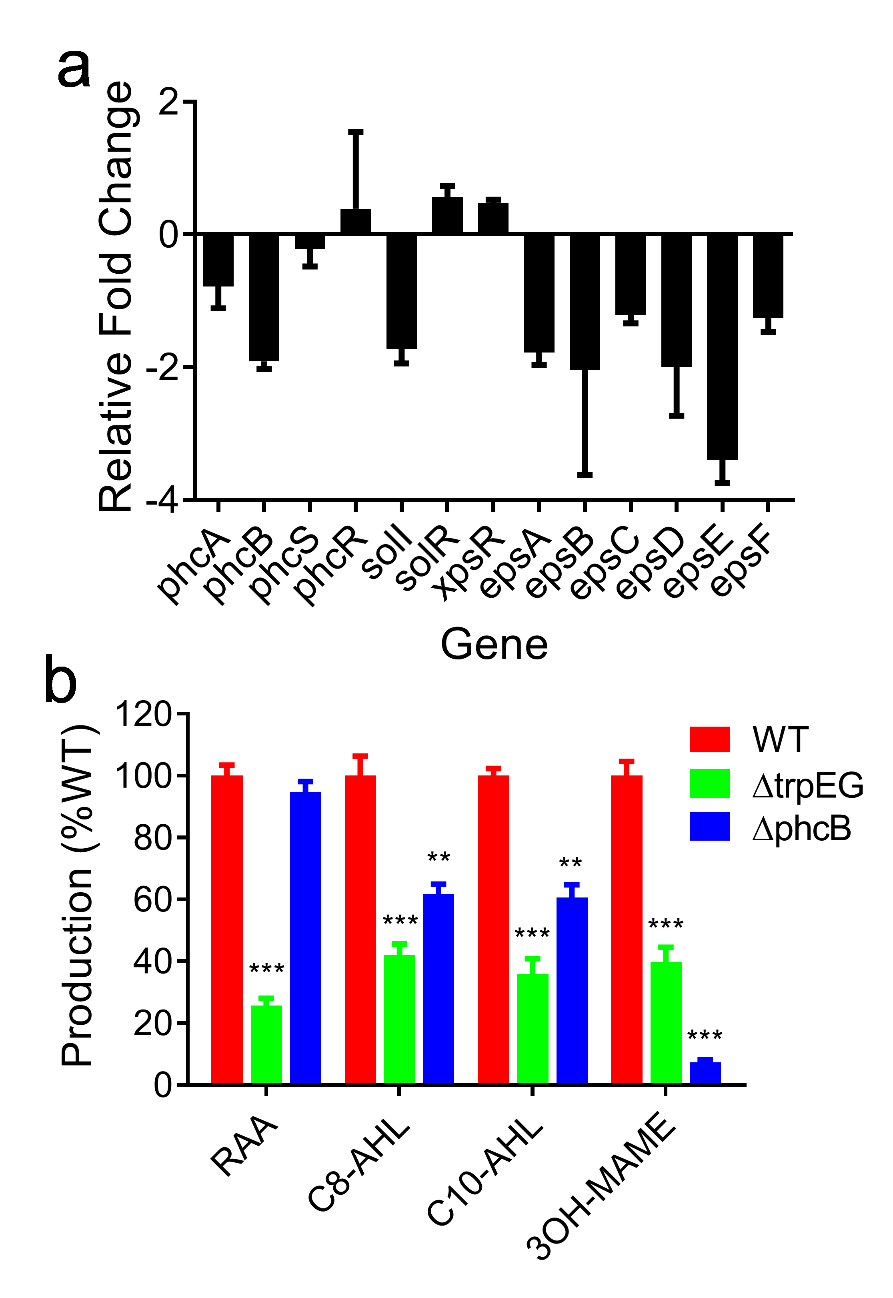
*
