# Supplementary material for: Anthranilic acid from Ralstonia solanacearum plays dual roles in intraspecies signalling and inter-kingdom communication
Source: ISME J. 2020 May 26;14(9):2248–60. doi: 10.1038/s41396-020-0682-7 (PMC7608240; doi:10.1038/s41396-020-0682-7)
Supplement: Supplementary file 17 — Supplementary Figure 15 [file 41396_2020_682_MOESM17_ESM.docx]

**Supplementary Figure 15** Analysis of DHQ by LC-MASS. (a) HPLC chromatograms of the standard DHQ. (b) ESI-MS spectra of the standard DHQ. (c) HPLC chromatograms of the ethyl acetate extract of *R. solanacearum* GMI1000. (d) ESI-MS spectra of the ethyl acetate extract of *R. solanacearum* GMI1000.

*
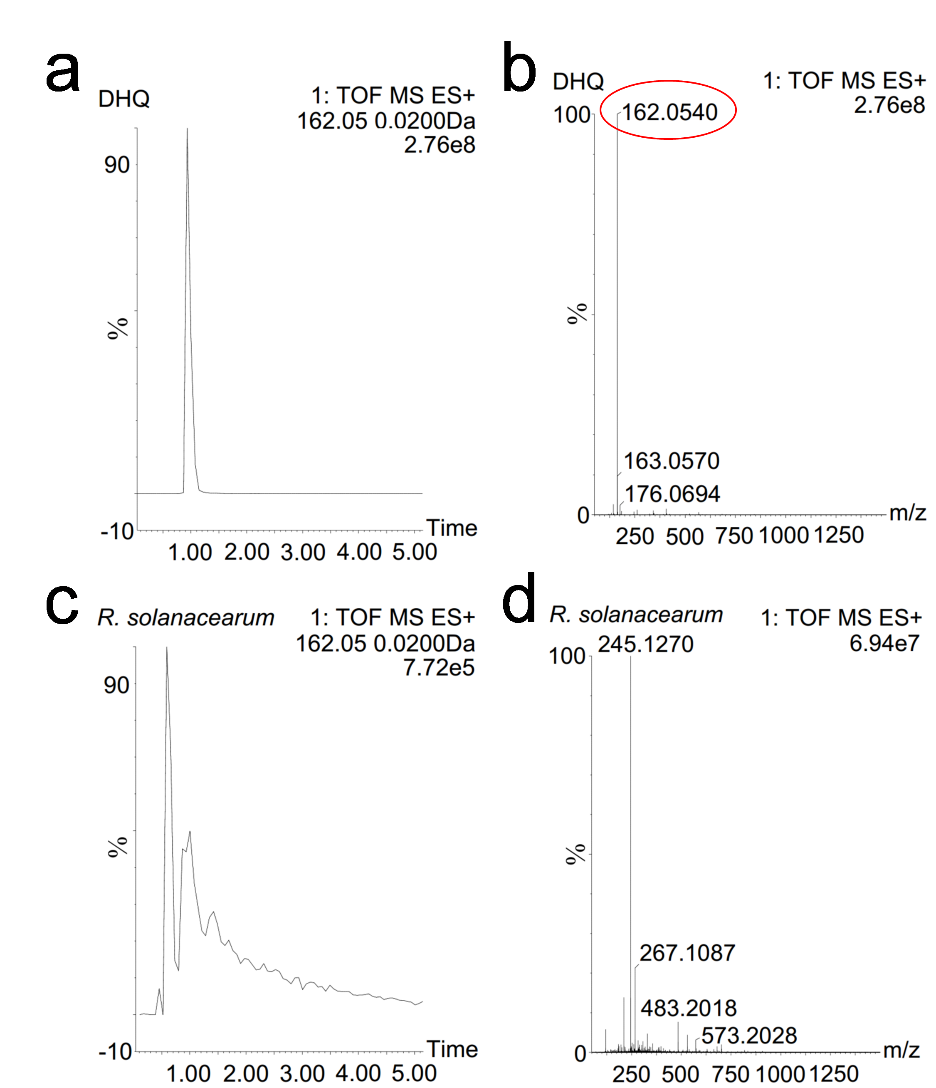
*
