# Supplementary material for: Anthranilic acid from Ralstonia solanacearum plays dual roles in intraspecies signalling and inter-kingdom communication
Source: ISME J. 2020 May 26;14(9):2248–60. doi: 10.1038/s41396-020-0682-7 (PMC7608240; doi:10.1038/s41396-020-0682-7)

**Supplementary Figure 16** Effect of the addition of exogenous PQS, HHQ and DHQ on the phenotypes of motility (a), biofilm formation (b), EPS production (c) and cellulase production (d) in the ∆trpEG mutant strain. The data are means ± standard deviations of three independent experiments. ***p < 0.001 (unpaired t-test).


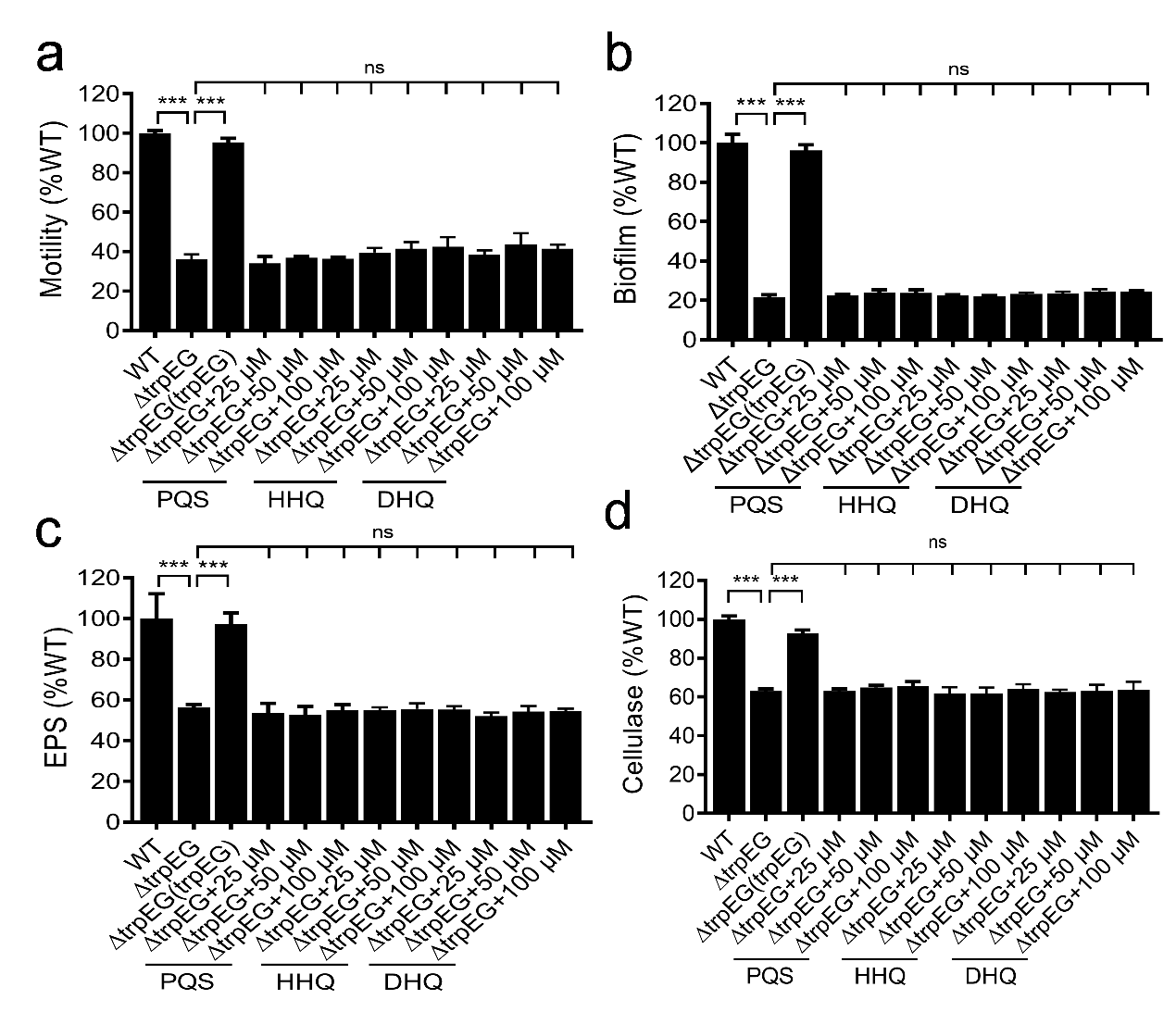

Supplement: Supplementary file 18 — Supplementary Figure 16 [file 41396_2020_682_MOESM18_ESM.docx]
