# Supplementary material for: Anthranilic acid from Ralstonia solanacearum plays dual roles in intraspecies signalling and inter-kingdom communication
Source: ISME J. 2020 May 26;14(9):2248–60. doi: 10.1038/s41396-020-0682-7 (PMC7608240; doi:10.1038/s41396-020-0682-7)
Supplement: Supplementary file 20 — Supplementary Figure 18 [file 41396_2020_682_MOESM20_ESM.docx]

**Supplementary Figure 18** Amino acid sequence alignment of TrpEG homologues of various bacterial species. Dark and blue coloration was used to label conserved sequences.

*
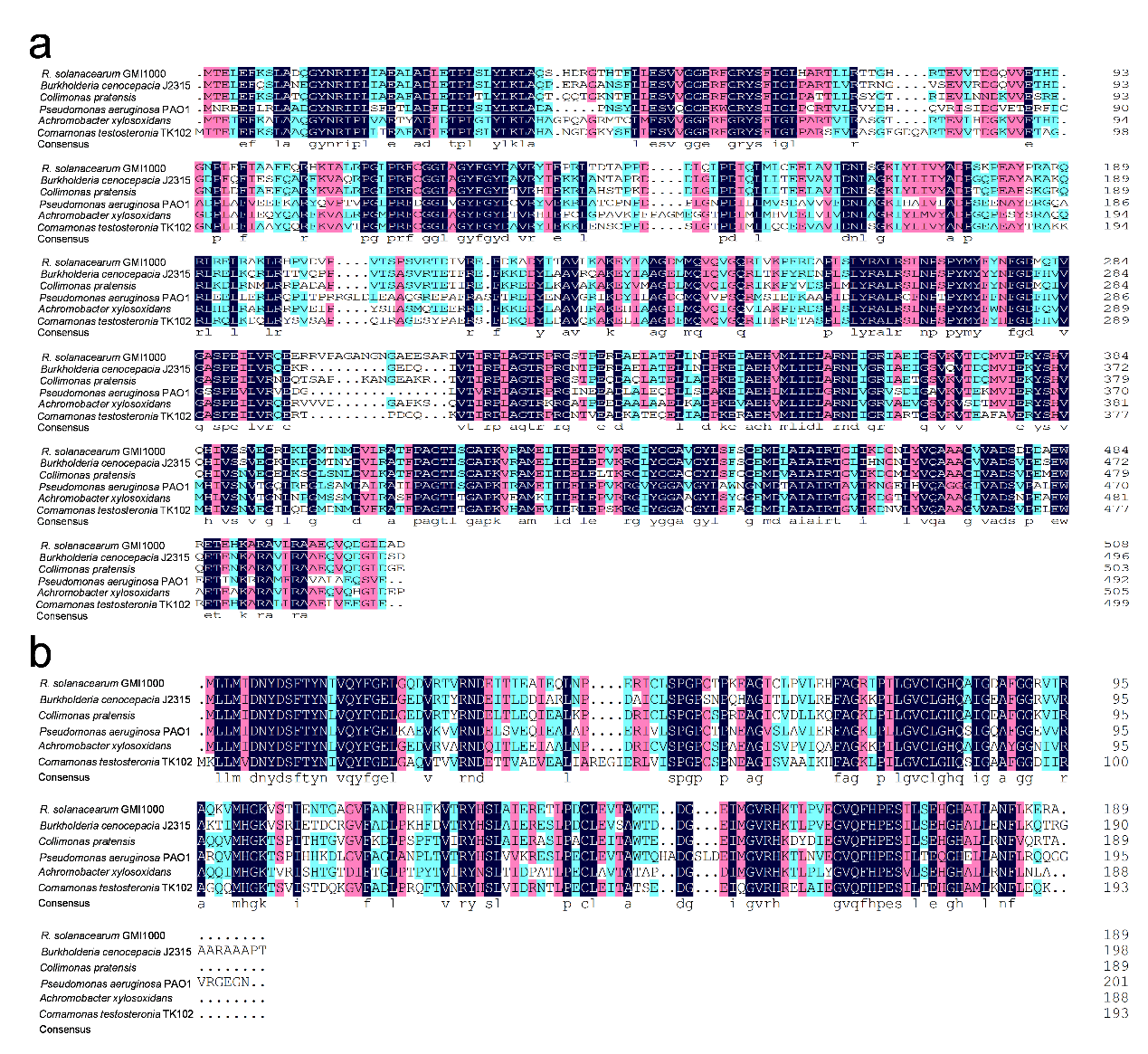
*
