# Supplementary material for: Anthranilic acid from Ralstonia solanacearum plays dual roles in intraspecies signalling and inter-kingdom communication
Source: ISME J. 2020 May 26;14(9):2248–60. doi: 10.1038/s41396-020-0682-7 (PMC7608240; doi:10.1038/s41396-020-0682-7)
Supplement: Supplementary file 21 — Supplementary Figure 19 [file 41396_2020_682_MOESM21_ESM.docx]

**Supplementary Figure 19** Analysis of anthranilic acid production in various bacteria by LC-MASS. (a) HPLC chromatograms of anthranilic acid in different bacteria. (b) ESI-MS spectra of anthranilic acid in different bacteria.

*
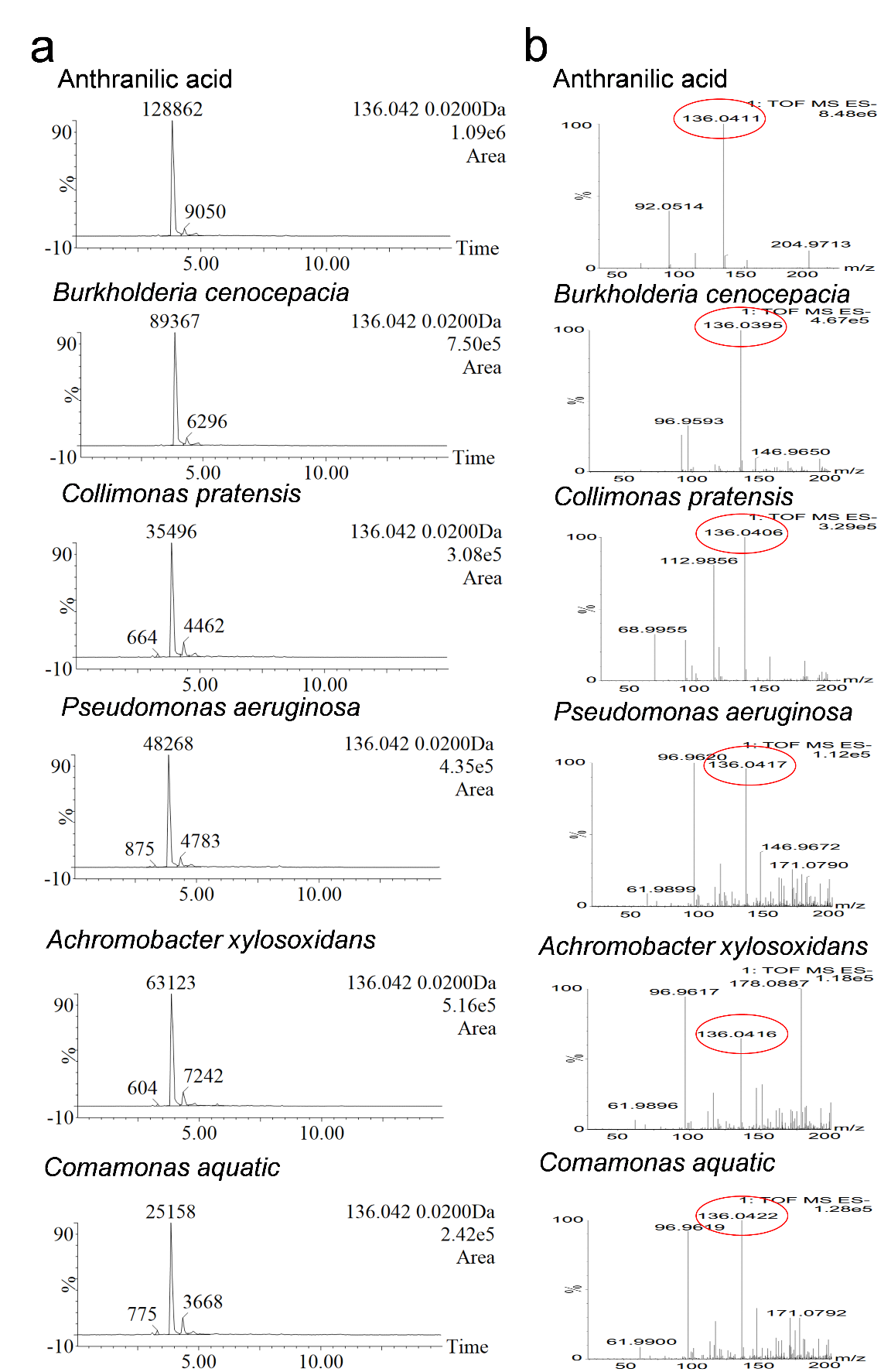
*
