# Supplementary material for: Anthranilic acid from Ralstonia solanacearum plays dual roles in intraspecies signalling and inter-kingdom communication
Source: ISME J. 2020 May 26;14(9):2248–60. doi: 10.1038/s41396-020-0682-7 (PMC7608240; doi:10.1038/s41396-020-0682-7)
Supplement: Supplementary file 22 — Supplementary Table legends [file 41396_2020_682_MOESM22_ESM.docx]

**Supplementary Table legends**

**Supplementary Table 1** Bacterial strains and plasmids used in this study.

**Supplementary Table 2** PCR primers used in this study.

**Supplementary Table 3** ^1^H (500 MHz) and ^13^C (125 MHz) NMR data of anthranilic acid (*δ* in ppm).

**Supplementary Table 4** List of genes differentially expressed in the *trpEG* mutant compared to the wild-type strain (Log_2_-fold change ≥ 1.5). Significantly differentially expressed genes were determined by Cufflinks after Benjamini-Hochberg correction. The fold-change is the ratio of the mutant FPKM to the wild-type FPKM.

**Supplementary Table 5** Analysis of the homologues of *trpE* and *trpG* in various bacterial species.
