# Supplementary material for: Anthranilic acid from Ralstonia solanacearum plays dual roles in intraspecies signalling and inter-kingdom communication
Source: ISME J. 2020 May 26;14(9):2248–60. doi: 10.1038/s41396-020-0682-7 (PMC7608240; doi:10.1038/s41396-020-0682-7)
Supplement: Supplementary file 23 — Supplementary Table 1 [file 41396_2020_682_MOESM23_ESM.docx]

**Supplementary Table 1** Bacterial strains and plasmids used in this study

| **Strain or plasmid** | **Phenotype and/or characteristic(s)^a^** | **Source or reference** |
| --- | --- | --- |
| ***R. solanacearum*** |  |  |
| GMI1000 | Wild-type strain of *R.* *solanacearum* | ATCCBAA-1114 |
| ΔtrpEG | Anthranilic acid-minus mutant derived from GMI1000 with *trpEG* being deleted | This study |
| ΔtrpE | Anthranilic acid-minus mutant derived from GMI1000 with *trpE* being deleted | This study |
| ΔtrpG | Anthranilic acid-minus mutant derived from GMI1000 with *trpG* being deleted | This study |
| ΔtrpEG(trpEG) | Mutant ΔtrpEG harboring the expression construct pLAFR3-*trpEG* | This study |
| ΔtrpE(trpE) | Mutant ΔtrpE harboring the expression construct pLAFR3-*trpE* | This study |
| ΔtrpG(trpG) | Mutant ΔtrpG harboring the expression construct pLAFR3-*trpG* | This study |
| ΔkynAUB | Mutant derived from GMI1000 with *kynAUB* being deleted | This study |
| ΔtrpEGΔkynAUB | Mutant derived from GMI1000 with *trpEG* and *kynAUB* being deleted | This study |
| GMI1000(P*trpEG*-*lacZ*) | GMI1000 harboring the reporter construct P*trpEG*-*lacZ* | This study |
| ΔtrpEG(P*trpEG*-*lacZ*) | ΔtrpEG harboring the reporter construct P*trpEG*-*lacZ* | This study |
| GMI1000(P*epsA*-*lacZ*) | GMI1000 harboring the reporter construct P*epsA*-*lacZ* | This study |
| ΔtrpEGΔkynAUB (P*epsA*-*lacZ*) | ΔtrpEGΔkynAUB harboring the reporter construct P*epsA*-*lacZ* | This study |
| ***E. Coli*** |  |  |
| DH5α | *supE44 lacU169(80lacZ M15) hsdR17 recA1 endA1 gyrA96 thi-1 relA1 pir* | Laboratory collection |
| BL21 | *F-ompT hsdS (rB-mB-) dcm+ Tetr gal (DE3) endA* | Stratagene |
| ***S. scitamineum*** |  |  |
| MAT-1 | Pair of mating strains of *S. scitamineum* | Yan et al. [1] |
| MAT-2 | Pair of mating strains of *S. scitamineum* | Yan et al. [1] |
| MAT-1 GFP | GFP-tagged strains of *S. scitamineum* | Yan et al. [1] |
| MAT-2 RFP | RFP-tagged strains of *S. scitamineum* | Yan et al. [1] |
| **Plasmid** |  |  |
| pME2-*LacZ* | Broad-host-range cloning vector, Tet^r^ | Kovach et al. [2] |
| P*trpEG*-*LacZ* | pME2-*lacZ* containing the promoter of *trpEG*, Tet^r^ | This study |
| P*epsA*-*LacZ* | pME2-*lacZ* containing the promoter of *epsA*, Tet^r^ | This study |
| pK18 | pK18, *sacB*^+^; gene replacement vector, Kan^r^ | Laboratory collection |
| pK18-*trpEG* | pK18 containing fragments flanking *trpEG*, Kan^r^ | This study |
| pK18-*trpE* | pK18 containing fragments flanking *trpE*, Kan^r^ | This study |
| pK18-*trpG* | pK18 containing fragments flanking *trpG*, Kan^r^ | This study |
| pK18-*kynAUB* | pK18 containing fragments flanking *kynAUB*, Kan^r^ | This study |
| pLAFR3 | Broad host range cloning vector, Tet^r^ | Staskawicz et al. [3] |
| pLAFR3-*trpEG* | pLAFR3 containing *trpEG*, Tet^r^ | This study |
| pLAFR3*-trpE* | pLAFR3 containing *trpE*, Tet^r^ | This study |
| pLAFR3-*trpG* | pLAFR3 containing *trpG*, Tet^r^ | This study |
| pET28a | Expression vector, Kan^r^ | Novagen |
| pET28a-*trpEG* | pET28a containing *trpEG*, Kan^r^ | This study |

^a^ Trim^r^, Kan^r^, Tet^r^, Amp^r^, Gm^r^ and Cat^r^ indicate resistance to trimethoprim, kanamycin, tetracycline, ampicillin, gentamicin and chloramphenicol, respectively.

**Supplementary References**

1. Yan M, Cai E, Zhou J, Chang C, Xi P, Shen W, et al. A dual-color imaging system for sugarcane smut fungus *Sporisorium scitamineum*. *Plant Dis.* 2016;100:2357-2362.
2. Kovach ME, Elzer PH, Hill DS, Robertson GT, Farris MA, Roop RM, Peterson, KM. Four new derivatives of the broad-host-range cloning vector pbbr1mcs, carrying different antibiotic-resistance cassettes. *Gene*. 1995;166:175-176.
3. Staskawicz B, Dahlbeck D, Keen N, Napoli C. Molecular characterization of cloned avirulence genes from race 0 and race 1 of *Pseudomonas syringae* pv. g*lycinea*. *J. Bacteriol.* 1987;169:5789–5794.
