# Supplementary material for: Anthranilic acid from Ralstonia solanacearum plays dual roles in intraspecies signalling and inter-kingdom communication
Source: ISME J. 2020 May 26;14(9):2248–60. doi: 10.1038/s41396-020-0682-7 (PMC7608240; doi:10.1038/s41396-020-0682-7)
Supplement: Supplementary file 24 — Supplementary Table 2 [file 41396_2020_682_MOESM24_ESM.docx]

**Supplementary Table 2** PCR primers used in this study

| **Primer** | **Sequence (5’-3’)** |
| --- | --- |
| For deletion |  |
| trpEGL-F | CGGGATCCCCTGATGTTCTGCTAACGT |
| trpEGL-R | TAGGCAAATGCCGATGTACTCCTTGGCCTTGAG |
| trpEGR-F | GAGTACATCGGCATTTGCCTACCGGTGCTG |
| trpEGR-R | CCAAGCTTATGATCTCGCCGTCTTCGGT |
| trpEL-F | CGCGGATCCAACTGACGATCCATGTTCCT |
| trpEL-R | CCTTGATGATAACTCGAATTCAAATCGCTG |
| trpER-F | AATTCGAGTTATCATCAAGGACGGCAACCT |
| trpER-R | CCCAAGCTTAATTGCTCGATCGCCTCGAT |
| trpGL-F | CGCGGATCCCTCAAGGCCAAGGAGTACAT |
| trpGL-R | TCAGGAAATTGGAGTCGTAGTTGTCGATCA |
| trpGR-F | CTACGACTCCAATTTCCTGAAGGAACGCGC |
| trpGR-R | CCCAAGCTTATGGTCGTGGGATTCGGTTA |
| kynAUBL-F | CCGGAATTCTTGGAACGTACTTTGTGGGA |
| kynAUBL-R | GCGATCAGAGACTGTGGGGCAGTGAAAGC |
| kynAUBR-F | GCCCCACAGTCTCTGATCGCTGCGTCTTAT |
| kynAUBR-R | CGCGGATCCGCAAGTACGGGCTCGACG |
| For *in trans* expression |  |
| trpEG-F | CCCAAGCTTATGACCGAACTCGAATTCAA |
| trpEG-R | CGCGGATCCTCAGGCGCGTTCCTTCAG |
| trpE-F | CCCAAGCTTATGACCGAACTCGAATTCAA |
| trpE-R | CGCGGATCCCTGTCAGAAATCGGCGTCCA |
| trpG-F | CCCAAGCTTATGCTGCTGATGATCGACAA |
| trpG-R | CGCGGATCCTCAGGCGCGTTCCTTCAG |
| For protein expression |  |
| trpEG-His-F | CCCAAGCTTATGACCGAACTCGAATTCAA |
| trpEG-His-R | CGCGGATCCTCAGGCGCGTTCCTTCAG |
| For reporter |  |
| p*trpEG*-F | CCCAAGCTTAACAAGCCGCACGATTTCAC |
| p*trpEG*-R | CCGCTCGAGGATGAAGGAATAGCGCCCGA |
| p*epsA*-F | CCGCTCGAGCCTGCCAGCCTGTAACGC |
| p*epsA*-R | CCGCTCGAGCACGCTCAACGACACGACT |
| For qPCR |  |
| RS_RS21330-F | TCCTCAACATCATCGCAGCC |
| RS_RS21330-R | TGCATCGGATCCTGGTTGTT |
| RS_RS21775-F | CCACGGACACAACAAGGTGA |
| RS_RS21775-R | AGGTTGACGGTCTCGATGTC |
| narH-F | TCGACAGTCTGAACAAGGGC |
| narH-R | TTGCTTTGCTCGATCATGCG |
| RS_RS26305-F | GGAGGTGCCATGTTCAAAGC |
| RS_RS26305-R | GCGGAAAGGGATATTTGGCG |
| RS_RS05515-F | TGCATGTCGCACTCGAACTC |
| RS_RS05515-R | AGCGCCTCGACAAACTGAG |
| RS_RS05760-F | AGTCGGGCGTGAAGAAATCG |
| RS_RS05760-R | CGATGAGGTGACGTCCTTGG |
| RS_RS05945-F | CATGCTCGGCGAATGTTCAG |
| RS_RS05945-R | TTCTCGTAGCCGAACAGCTC |
| RS_RS06385-F | CTTCGTTCTTGCCGATACGC |
| RS_RS06385-R | TAGGCACGCTTATCTTCGGG |
| ccoN-F | ACCTACAGCTTTGTCGAGGC |
| ccoN-R | GAGGGTCTTGAGCACGTTGA |
| ccoO-F | CGCTACGGCCATTACTCGAT |
| ccoO-R | CGCATCTTCGCTTCGATGTC |
| ccoP-F | TGGATGTTCCTGCTGTCGTG |
| ccoP-R | GCGCAGTTGTTGAGGAACAG |
| RS_RS06485-F | GGGTGGACCAGTTCAACGAC |
| RS_RS06485-R | GCTGGATCTTCGACTCGGTG |
| RS_RS06490-F | GAAAGCGTGGGCTACCTCCT |
| RS_RS06490-R | GATGTCGGTGTTCAGTTCCC |
| RS_RS06710-F | GACATCCAGCCCGTCTACCT |
| RS_RS06710-R | GTGTTGTTGGACGACAGCC |
| RS_RS07450-F | CGGAACACCATCCGCTGT |
| RS_RS07450-R | AGCAGGGTCAGATTGATGACG |
| RS_RS07510-F | GCGATTGAAGTACCGCTTCG |
| RS_RS07510-R | CTTCTGGACCCTGCGTTCAA |
| motA-F | GCTAGTCGCCATCGGTTACA |
| motA-R | GATCGCCTTCTTGTCGTTGC |
| RS_RS07520-F | AATTTCTGGATCACGCCGGA |
| RS_RS07520-R | GGTTCTGGCGGTAGGGAATG |
| RS_RS07560-F | GAGGTCGAGGCACTGTTTGT |
| RS_RS07560-R | GCGACGACGGTTTTTCCATC |
| RS_RS08835-F | TCGCCTACATCGTCAACCAC |
| RS_RS08835-R | CAGCAGGTCTTCGTAGCACA |
| RS_RS09495-F | CGACAACTCGAAGCCTTTGC |
| RS_RS09495-R | GGTGTGTTCGACGTCCTCAT |
| RS_RS10880-F | CCTGCTGATCGTCTACCTGG |
| RS_RS10880-R | GAAGAACAGGCCGACGTTGT |
| RS_RS25270-F | TGCCTCAGAACGAACGAAGA |
| RS_RS25270-R | TCAGCCATCACTGTCGGAATC |
| RS_RS12710-F | CTGTCGGATCGGTTGCACTC |
| RS_RS12710-R | GACAATCGTGATCCACCAGC |
| RS_RS12975-F | AGCGCATTCCCTTGGAAGAA |
| RS_RS12975-R | GAGCGCCATCATCAAATCGC |
| RS_RS13255-F | ATCTCGTCAACGGCTACCTG |
| RS_RS13255-R | CGTAGTCGACGGTCGGAATG |
| epsF-F | TGCGTTCTACGAGTTCCAGC |
| epsF-R | TTGGCCACGGAATACGAGAG |
| RS_RS14005-F | GCGTGATTTCGAGCGCAAG |
| RS_RS14005-R | GCGATGGGAATCTTGGTTTCG |
| RS_RS15320-F | AGGCTGATGACCTTCGACAA |
| RS_RS15320-R | GTAGCGCGAGAAGGCATAGG |
| RS_RS01575-F | CTCCCGTCATACCCTGCTG |
| RS_RS01575-R | TAACCGTAGGTATCGGCCTG |
| RS_RS16495-F | CGGGTCAAGAGAATCGTGCT |
| RS_RS16495-R | CATCACGGCGACAAAGAACC |
| RS_RS16525-F | TTTTACTGGGACATCCGCCC |
| RS_RS16525-R | CCAGGTAATCGTCCTCGGTG |
| cel-F | CTGCTCGATCCGCACAACTA |
| cel-R | ATTGCCCTTGAACTGGGTGG |
| cheW-F | CGAGGAATACGGCATCGACA |
| cheW-R | GACGACGGTGTACTGGTTGT |
| RS_RS16530-F | TCGTGGTGATGATCGTGTCG |
| RS_RS16530-R | TATCGTGCATCAGCAGCCAG |
| RS_RS16970-F | GCTGCCATCAACACCGACTA |
| RS_RS16970-R | CGCACCTCTTCCGACTGATA |
| treZ-F | TCTCGTCGCTGTTCCTGTTC |
| treZ-R | AAGTGATACAGCACGGTCGG |
| RS_RS18605-F | CCAGGCGAATCTCAACGGTA |
| RS_RS18605-R | TGCCTTCGATGTCTTCCGAC |
| RS_RS18840-F | CTGTCGGTGGGCTACAAGAG |
| RS_RS18840-R | TGCCCTCTTTGTTCTGCGTA |
| flgL-F | TCAGTACCGCCCAGTTCTTC |
| flgL-R | GTCATTCGTGTCCTGCGACT |
| RS_RS18995-F | CTGACCACGACCCTGAACTC |
| RS_RS18995-R | ATGTTCTTGTTGGCCGTTGC |
| RS_RS19000-F | GCATTTCGCTGAACTCGCTC |
| RS_RS19000-R | CCATCGACTGATAGGTGCCC |
| RS_RS19095-F | GGTGAACGGCAAGTTCGACA |
| RS_RS19095-R | ACCGTCCGTGTATGGGTC |
| RS_RS19560-F | TGCTGACATCCACGCTCAAT |
| RS_RS19560-R | AACGCGAGCCATAAAATGCC |
| RS_RS20835-F | TGAGGGTGCTTTTTGGCGAT |
| RS_RS20835-R | CGCGATCAAATGCTTCGTGT |
| RS_RS20855-F | ATGACCAACCTCATCACCGAT |
| RS_RS20855-R | CGGACATCGAAGCGACTCTC |
| phcA-F | CTTCAACATCAGCTTCGCCG |
| phcA-R | TCCAGCTCATTGGAACGCAT |
| phcB-F | TATCGCACCTACACCAAGCC |
| phcB-R | CGCCGAGATAGTTGACCAGG |
| phcS-F | CGCCAACTACCAGGGCTATC |
| phcS-R | GGTCAGCATCATCAGCGAGA |
| phcR-F | GTTTCTCGCCCACGAACTGA |
| phcR-R | CGCACCGAATTCAGGAAGGT |
| solI-F | CGGCCCTATCTGCTCAAGG |
| solI-R | AAGGTCACGCCGATCAGTC |
| solR-F | CCAGCTTTTTCGGCAGATCG |
| solR-R | CCTCATCACGGATGGACACC |
| xpsR-F | CAGAGCGAATGCGACTGGAA |
| xpsR-R | TCCGGCACAGATCGACATAG |
| epsA-F | CATTACGCCCGATCTTGTGC |
| epsA-R | AATAGGTCTGCGTCGGGAAC |
| epsB-F | TCGCTGGAAGGCAAGAATCC |
| epsB-R | GCAGATTGCGCATCTCGTTG |
| epsC-F | TCCTGGTTGTATTCGGTACGC |
| epsC-R | CCCGTCAGGATATTGCTGGT |
| epsD-F | GACCTGTCGTACATCGAGGC |
| epsD-R | CGGATATCGGACTCTTCGCC |
| epsE-F | ATCACCTTCATCCCGGCAAC |
| epsE-R | GCTGGATAAAGCCACGCAAA |
| epsF-F | GGCCTTTCTGCTTTTCGTCG |
| epsF-R | CCCTGGTATCTGGCACTCAC |
| G16S-F | AGGCCTTCGGGTTGTAAAG |
| G16S-R | CGTAGTTAGCCGGTCCTTATTC |
