# Supplementary material for: Anthranilic acid from Ralstonia solanacearum plays dual roles in intraspecies signalling and inter-kingdom communication
Source: ISME J. 2020 May 26;14(9):2248–60. doi: 10.1038/s41396-020-0682-7 (PMC7608240; doi:10.1038/s41396-020-0682-7)
Supplement: Supplementary file 25 — Supplementary Table 3 [file 41396_2020_682_MOESM25_ESM.docx]

**Supplementary Table 3** ^1^H (500 MHz) and ^13^C (125 MHz) NMR data of anthranilic acid (*δ* in ppm)

| Position | Anthranilic acid (in CD_3_OD) | | |
| --- | --- | --- | --- |
|  | *δ* (H) | *δ* (C) |  |
| 1 |  | 110.31 |  |
| 2 |  | 151.38 |  |
| 3 |  | 116.33 |  |
| 4 | 6.74(1H,m), | 133.60 |  |
| 5 | 7.22(1H,m), | 115.17 |  |
| 6 | 6.5(1H,m) | 131.25 |  |
| 7 | 7.81(1H,m) | 170.25 |  |
