# Supplementary material for: Anthranilic acid from Ralstonia solanacearum plays dual roles in intraspecies signalling and inter-kingdom communication
Source: ISME J. 2020 May 26;14(9):2248–60. doi: 10.1038/s41396-020-0682-7 (PMC7608240; doi:10.1038/s41396-020-0682-7)
Supplement: Supplementary file 26 — Supplementary Table 4 [file 41396_2020_682_MOESM26_ESM.docx]

**Supplementary Table 4** List of genes differentially expressed in the *trpEG* mutant compared to the wild-type strain (Log_2_-fold change ≥ 1.5). Significantly differentially expressed genes were determined by Cufflinks after Benjamini-Hochberg correction. The fold-change is the ratio of the mutant FPKM to the wild-type FPKM.

| **Class** | **Gene ID^a^** | **Fold change** | **Description** |
| --- | --- | --- | --- |
| Flagella synthesis, attachment, motility and chemotaxis | RS_RS05755 | -1.68 | chemotaxis protein |
|  | RS_RS05760 | -2.65 | methyl-accepting chemotaxis protein |
|  | RS_RS19040 | -1.75 | flagellar motor switch protein FliG |
|  | RS_RS23695 | -2.97 | methyl-accepting chemotaxis protein |
|  | RS_RS23885 | -1.58 | protein phosphatase CheZ |
|  | RS_RS23890 | -1.64 | response regulator |
|  | RS_RS23895 | -2.90 | chemotaxis response regulator protein-glutamate methylesterase |
|  | RS_RS23900 | -1.91 | probable chemotaxis protein |
|  | RS_RS23905 | -2.52 | chemotaxis protein CheR |
|  | RS_RS23915 | -2.43 | chemotaxis protein CheW |
|  | RS_RS23925 | -1.59 | response regulator |
|  | *motA* | -1.80 | flagellar motor protein MotA |
|  | RS_RS18840 | -2.20 | flagellar hook-associated protein FlgK |
|  | *flgL* | -2.45 | flagellar hook-associated protein 3 |
|  | *fliQ* | -2.66 | flagellar export apparatus protein FliQ |
|  | *fliP* | -2.27 | flagellar biosynthetic protein FliP |
|  | *flhF* | -1.53 | flagellar biosynthesis regulator FlhF |
|  | RS_RS18995 | -4.10 | flagellin |
|  | RS_RS19000 | -2.40 | flagellar hook protein FliD |
|  | *fliS* | -2.02 | flagellar protein FliS |
|  | RS_RS19045 | -1.77 | flagellar assembly protein FliH |
|  | *fliJ* | -1.85 | flagellar export protein FliJ |
| Membrane components and transporters | RS_RS00425 | -2.61 | amino acid ABC transporter substrate-binding protein |
|  | RS_RS04575 | 1.79 | methionine import ATP-binding protein MetN |
|  | RS_RS06700 | 2.41 | ABC transporter permease |
|  | RS_RS06710 | 2.05 | sulfonate ABC transporter substrate-binding protein |
|  | RS_RS06720 | 2.77 | sulfonate ABC transporter |
|  | RS_RS06725 | 1.91 | aliphatic sulfonate ABC transporter ATP-binding protein |
|  | *cysT* | 3.22 | sulfate ABC transporter permease subunit CysT |
|  | RS_RS06915 | -1.75 | ABC transporter ATP-binding protein |
|  | RS_RS06920 | -2.68 | glutathione ABC transporter substrate-binding protein |
|  | RS_RS06925 | -2.35 | glutathione ABC transporter permease GsiC |
|  | RS_RS06930 | -1.69 | glutathione ABC transporter permease GsiD |
|  | RS_RS08840 | -2.37 | branched-chain amino acid ABC transporter substrate-binding protein |
|  | RS_RS11350 | 1.97 | amino acid ABC transporter substrate-binding protein |
|  | RS_RS12235 | -1.73 | ABC transporter ATP-binding protein |
|  | RS_RS12240 | -1.58 | ABC transporter ATP-binding protein |
|  | RS_RS12255 | -1.96 | branched chain amino acid ABC transporter substrate-binding protein |
|  | *pstS* | 1.93 | phosphate ABC transporter substrate-binding protein PstS |
|  | RS_RS16745 | -1.97 | ABC transporter permease |
|  | RS_RS16970 | 2.20 | metal ABC transporter substrate-binding protein |
|  | RS_RS20175 | -1.51 | ABC transporter ATP-binding protein |
|  | RS_RS23795 | 2.06 | sulfonate ABC transporter substrate-binding protein |
|  | RS_RS24755 | 4.02 | branched chain amino acid ABC transporter substrate-binding protein |
|  | *xylF* | -1.80 | D-xylose ABC transporter substrate-binding protein |
|  | *gspG* | 1.98 | type II secretion system protein GspG |
|  | RS_RS21245 | -2.44 | EscR/YscR/HrcR family type III secretion system export apparatus protein |
|  | RS_RS15330 | -3.65 | ABC transporter substrate-binding protein |
|  | RS_RS15305 | -2.33 | ABC transporter |
|  | RS_RS15315 | -2.29 | ABC transporter permease |
|  | RS_RS15310 | -2.17 | ABC transporter ATP-binding protein |
|  | RS_RS15320 | -1.98 | sugar ABC transporter permease |
|  | RS_RS21780 | -14.75 | zinc-binding protein |
|  | RS_RS20855 | -5.24 | phosphopantetheine-binding and acyl carrier protein |
|  | RS_RS21145 | -2.10 | peptidoglycan-binding protein LysM |
|  | RS_RS04325 | -1.90 | DNA-binding protein |
|  | RS_RS20575 | -1.72 | FMN-binding protein |
|  | RS_RS06420 | -12.21 | membrane protein |
|  | RS_RS14870 | -8.05 | membrane protein |
|  | RS_RS20875 | -4.77 | membrane protein |
|  | RS_RS06425 | -3.21 | probable transmembrane protein |
|  | RS_RS04355 | -3.93 | probable transmembrane protein |
|  | RS_RS21800 | -3.41 | probable transmembrane protein |
|  | RS_RS19745 | -2.80 | probable transmembrane protein |
|  | RS_RS06485 | -2.74 | outer membrane CHANEL lipoprotein |
|  | RS_RS10875 | -2.35 | membrane protein |
|  | RS_RS15325 | -2.33 | membrane protein |
|  | RS_RS13650 | -2.15 | membrane protein |
|  | RS_RS18670 | -2.13 | membrane protein |
|  | RS_RS10880 | -2.03 | membrane protein |
|  | RS_RS07450 | -2.31 | probable transmembrane protein |
|  | RS_RS12175 | 2.92 | anion permease |
|  | RS_RS23260 | -3.00 | anion permease |
|  | RS_RS23240 | -1.75 | anion permease |
|  | RS_RS25155 | 1.60 | adenine permease |
|  | RS_RS25270 | -2.56 | probable pseudogene (type III effector protein, avrpm1 homologue) |
|  | RS_RS26630 | -2.33 | type III effector protein (plasmid) |
|  | RS_RS18605 | -1.90 | probable avrpphd family type III effector protein |
|  | RS_RS22065 | 2.52 | Type VI secretion system (T6SS), amidase immunity protein |
|  | RS_RS19630 | 2.34 | mechanosensitive ion channel protein MscS |
|  | RS_RS10175 | -2.24 | lipoprotein transmembrane |
|  | RS_RS09330 | -2.23 | probable transmembrane protein |
|  | RS_RS00895 | -1.92 | probable transmembrane protein |
|  | RS_RS08970 | -1.87 | membrane protein |
|  | RS_RS00980 | -1.67 | probable transmembrane protein |
|  | RS_RS22975 | -1.58 | lipoprotein transmembran |
|  | RS_RS15950 | -1.56 | probable transmembrane protein |
|  | RS_RS26160 | -1.52 | membrane protein |
|  | RS_RS21855 | 1.50 | probable transmembrane protein |
|  | RS_RS17695 | 1.55 | membrane protein |
|  | RS_RS12385 | 1.55 | membrane protein |
|  | RS_RS16720 | 1.58 | membrane protein |
|  | RS_RS15880 | 1.91 | ATP-binding protein |
|  | RS_RS17820 | 1.58 | membrane protein |
|  | RS_RS19430 | 1.65 | hypothetical transmembrane protein |
|  | RS_RS24475 | 1.68 | rhomboid family intramembrane serine protease |
|  | RS_RS16495 | 1.69 | membrane protein |
|  | RS_RS19590 | 1.71 | membrane protein |
|  | RS_RS24995 | 1.79 | membrane protein |
|  | RS_RS25090 | 1.80 | membrane protein |
|  | RS_RS17700 | 1.81 | membrane protein |
|  | RS_RS26205 | 1.87 | probable transporter lipoprotein transmembrane |
|  | RS_RS22075 | 2.10 | membrane protein |
|  | RS_RS25130 | 2.13 | membrane protein (plasmid) |
|  | RS_RS25095 | 2.25 | membrane protein |
|  | RS_RS22780 | 2.33 | probable transmembrane protein |
|  | RS_RS19105 | 2.50 | probable transmembrane protein |
|  | RS_RS19120 | 2.90 | probable transmembrane protein |
|  | RS_RS21455 | 3.54 | probable transmembrane protein |
|  | RS_RS17850 | 3.82 | probable transmembrane protein |
|  | RS_RS21465 | 3.97 | transmembrane protein |
|  | RS_RS09345 | -2.06 | hypothetical transmembrane protein |
| signal peptide protein | RS_RS19095 | -3.64 | signal peptide protein |
|  | RS_RS24185 | -2.78 | probable signal peptide protein |
|  | RS_RS22120 | -1.89 | probable signal peptide protein |
|  | RS_RS24355 | -1.75 | probable signal peptide protein |
|  | RS_RS22755 | -1.72 | probable cog4727, uncharacterized conserved in bacteria signal peptide protein |
|  | RS_RS23265 | -1.72 | probable signal peptide protein |
|  | RS_RS20455 | -1.56 | dehydrogenase (Flavoproteins) signal peptide |
|  | RS_RS01680 | -1.52 | probable signal peptide protein |
|  | RS_RS17705 | 1.51 | signal peptide protein |
|  | RS_RS15340 | 1.57 | signal peptide protein |
|  | RS_RS20520 | 1.69 | probable signal peptide protein |
|  | RS_RS25045 | 1.84 | probable signal peptide protein |
|  | RS_RS14440 | 1.84 | signal peptide protein |
|  | RS_RS23115 | 1.93 | probable signal peptide protein |
|  | RS_RS04630 | 1.94 | probable signal peptide protein |
|  | RS_RS19995 | 2.10 | probable signal peptide protein |
|  | RS_RS22705 | 2.45 | probable signal peptide protein |
|  | RS_RS16915 | 2.58 | hypothetical signal peptide protein |
|  | RS_RS21470 | 4.72 | signal peptide protein |
|  | RS_RS25115 | 4.82 | signal peptide protein |
|  | RS_RS09370 | -7.83 | signal peptidase |
|  | RS_RS23970 | -3.00 | peptide synthetase |
|  | *prmC* | 1.51 | probable methylase of polypeptide chain release factors protein |
|  | RS_RS07530 | 1.58 | tetratricopeptide repeat family protein |
| Carbohydrate metabolism | RS_RS10685 | -1.61 | D-arabinitol 4-dehydrogenase |
|  | *galU* | 3.15 | UTP--glucose-1-phosphate uridylyltransferase |
|  | RS_RS21345 | -4.19 | polygalacturonase |
|  | RS_RS17735 | -1.57 | 2-methylcitrate synthase |
|  | *tal* | -1.58 | transaldolase |
|  | *pgl* | -1.65 | 6-phosphogluconolactonase |
|  | RS_RS24620 | -1.50 | phosphogluconate dehydratase |
|  | RS_RS10755 | -1.74 | D-tagatose-bisphosphate aldolase, class II, non-catalytic subunit |
|  | RS_RS13785 | -1.67 | gluconolaconase |
|  | RS_RS00960 | -1.87 | alcohol dehydrogenase |
|  | RS_RS15715 | -2.88 | aldehyde dehydrogenase |
|  | RS_RS26195 | -1.69 | glyoxalase i, nickel isomerase |
|  | RS_RS13685 | -1.53 | SAM-dependent methyltransferase PhcB |
|  | RS_RS13750 | -1.50 | transcriptional regulator PhcA |
|  | RS_RS22015 | -1.57 | EPS I polysaccharide export inner membrane protein EpsF |
|  | RS_RS22020 | -2.38 | EPS I polysaccharide export inner membrane protein EpsE |
|  | RS_RS22025 | -1.72 | UDP-N-acetyl-D-mannosamine dehydrogenase |
|  | RS_RS22030 | -1.65 | UDP-N-acetylglucosamine 2-epimerase (non-hydrolyzing) |
|  | RS_RS22035 | -1.53 | tyrosine protein kinase |
|  | RS_RS22045 | -1.79 | EPS I polysaccharide export outer membrane protein EpsA |
|  | *leuA* | 1.72 | 2-isopropylmalate synthase 2 |
|  | RS_RS16005 | 3.69 | glycoside hydrolase |
|  | RS_RS22310 | 2.14 | glucose-fructose oxidoreductase |
|  | RS_RS20890 | -5.03 | alpha/beta hydrolase |
|  | RS_RS20840 | -3.71 | alpha/beta hydrolase |
|  | RS_RS23965 | -3.49 | alpha/beta hydrolase |
|  | RS_RS22480 | -3.09 | alpha/beta hydrolase |
|  | RS_RS25245 | -1.69 | hydrolase |
|  | RS_RS08950 | -1.50 | alpha/beta hydrolase |
|  | RS_RS20445 | -2.09 | lactoylglutathione lyase |
|  | RS_RS06385 | -4.59 | copper-translocating P-type ATPase |
|  | RS_RS17915 | -1.52 | drug:proton antiporter |
|  | *treY* | 1.65 | malto-oligosyltrehalose synthase |
|  | *treZ* | 1.82 | malto-oligosyltrehalose trehalohydrolase |
|  | RS_RS07540 | 1.89 | 2,5-diketo-D-gluconate reductase A |
|  | RS_RS03570 | 2.29 | lytic transglycosylase |
|  | RS_RS18305 | 1.72 | glycogen-branching enzyme |
|  | *treS* | 1.67 | alpha-amylase |
|  | RS_RS18315 | 1.82 | alpha-amylase |
|  | RS_RS18320 | 2.91 | glycogen synthase |
|  | RS_RS02545 | 1.69 | mannose-1-phosphate guanylyltransferase |
|  | *otsB* | 1.65 | trehalose-phosphatase |
|  | RS_RS22700 | 2.27 | glycoprotein (plasmid) |
|  | *otsA* | 2.16 | trehalose-6-phosphate synthase |
|  | RS_RS24605 | -1.6 | glucokinase |
| Amino acid metabolism | *glyA* | 3.62 | serine hydroxymethyltransferase |
|  | RS_RS26005 | -3.16 | Serine/threonine protein kinase |
|  | RS_RS06935 | -2.24 | D-aminopeptidase |
|  | RS_RS11355 | 2.30 | D-amino acid dehydrogenase small subunit |
|  | RS_RS16810 | -2.11 | phenylalanine 4-monooxygenase |
|  | RS_RS20005 | -2.63 | phenylacetate-CoA oxygenase subunit PaaA |
|  | RS_RS20010 | -2.52 | phenylacetate-CoA oxygenase subunit PaaB |
|  | *paaI* | -1.96 | phenylacetic acid degradation protein |
|  | *paaJ* | -2.80 | phenylacetate-CoA oxygenase subunit PaaJ |
|  | RS_RS20025 | -2.64 | phenylacetic acid degradation protein |
|  | RS_RS21795 | -7.22 | peptidase (collagenase-like) |
|  | RS_RS21790 | -6.35 | U32 family peptidase |
|  | RS_RS04965 | -3.17 | peptidase S1 |
|  | RS_RS12975 | -2.08 | D-alanyl-D-alanine endopeptidase |
|  | RS_RS15405 | 1.50 | peptidase |
|  | RS_RS14180 | 1.88 | peptidase M23 |
|  | RS_RS16925 | 2.80 | phosphatidylserine synthase |
|  | RS_RS21400 | 2.21 | 2-keto-4-pentenoate hydratase |
|  | *hppD* | 1.56 | 4-hydroxyphenylpyruvate dioxygenase |
|  | RS_RS21395 | 2.06 | 2-hydroxymuconic semialdehyde dehydrogenase |
|  | RS_RS23700 | -3.24 | probable 2-oxoglutarate dehydrogenase e1 decarboxylase component oxidoreductase protein |
|  | RS_RS24725 | -2.41 | catalase |
|  | RS_RS02405 | -1.84 | class II glutamine amidotransferase |
|  | RS_RS13240 | -2.71 | imidazolonepropionase |
|  | RS_RS13245 | -2.97 | formimidoylglutamase |
|  | *hutH* | -3.16 | histidine ammonia-lyase |
|  | RS_RS13255 | -2.72 | urocanate hydratase |
|  | RS_RS00780 | 1.61 | arginase |
|  | *dapA* | 1.50 | 4-hydroxy-tetrahydrodipicolinate synthase |
|  | *putA* | 1.77 | trifunctional transcriptional regulator/proline dehydrogenase/L-glutamate gamma-semialdehyde dehydrogenase |
|  | RS_RS19480 | 1.67 | ornithine cyclodeaminase |
|  | RS_RS23450 | 2.33 | polyamine aminopropyltransferase 1 |
|  | RS_RS23580 | 2.33 | polyamine aminopropyltransferase 1 |
|  | RS_RS00470 | 1.67 | adenosylhomocysteinase |
|  | RS_RS16465 | -1.71 | acyl-homoserine-lactone synthase SolI |
|  | RS_RS16905 | 1.58 | 5'-methylthioadenosine/S-adenosylhomocysteine nucleosidase |
|  | RS_RS20210 | 1.52 | aminocyclopropane-1-carboxylate deaminase/D-cysteine desulfhydrase family protein |
|  | RS_RS20360 | 3.51 | 5-methyltetrahydropteroyltriglutamate--homocysteine methyltransferase |
|  | *gabT* | -3.49 | 4-aminobutyrate transaminase |
|  | RS_RS16525 | 1.98 | glutamate--cysteine ligase |
| Energy conversion | *ssuE* | 2.25 | FMN reductase (NADPH) |
|  | RS_RS18940 | 3.65 | 1-acyl-sn-glycerol-3-phosphate acyltransferase |
|  | RS_RS24495 | 2.37 | 2-oxoglutarate-dependent ethylene/succinate-forming enzyme |
|  | RS_RS06025 | -1.94 | NADPH:quinone oxidoreductase |
|  | RS_RS12160 | 1.76 | phosphoadenosine phosphosulfate reductase |
|  | RS_RS12170 | 2.06 | sulfite reductase |
|  | *cysC* | 2.07 | adenylyl-sulfate kinase |
|  | RS_RS12160 | 1.76 | phosphoadenosine phosphosulfate reductase |
|  | RS_RS20845 | -4.85 | cystathionine gamma-synthase |
|  | RS_RS09500 | 1.63 | FAD-dependent oxidoreductase |
|  | RS_RS24065 | 1.58 | probable serine acetyltransferase protein |
|  | RS_RS24745 | 3.58 | FAD-dependent oxidoreductase |
|  | RS_RS00560 | -5.13 | 2-nitropropane dioxygenase |
|  | RS_RS18665 | -2.22 | 2-nitropropane dioxygenase |
|  | RS_RS21805 | -3.52 | MFS transporter |
|  | RS_RS21810 | -3.77 | Nitrate/nitrite transporter (plasmid) |
|  | RS_RS21815 | -5.54 | nitrate reductase subunit alpha |
|  | *narH* | -2.00 | nitrate reductase 2 (NRZ), beta subunit |
|  | RS_RS23720 | -2.50 | TAT-dependent nitrous-oxide reductase |
|  | *nirK* | -5.31 | probable major anaerobically induced outer membrane transmembrane protein |
|  | *ccoN* | -7.85 | cytochrome c oxidase, cbb3-type subunit I |
|  | RS_RS21460 | 3.40 | dehydrogenase oxidoreductase |
|  | *ccoO* | -7.31 | peptidase S41 |
|  | RS_RS06405 | -13.23 | cytochrome oxidase |
|  | *ccoP* | -5.10 | probable cytochrome c oxidase (subunit III) transmembrane protein |
|  | *ccoS* | -13.06 | cytochrome oxidase maturation protein Cbb3 |
|  | RS_RS00985 | -2.77 | cytochrome b |
|  | RS_RS23715 | -2.43 | Cytochrome c-555 (plasmid) |
|  | *ccoG* | -2.22 | cytochrome c oxidase accessory protein CcoG |
|  | RS_RS01065 | -2.15 | short-chain dehydrogenase |
|  | RS_RS20465 | -1.77 | alcohol dehydrogenase |
|  | RS_RS10600 | -1.77 | oxidoreductase |
|  | RS_RS09225 | -1.60 | Indolepyruvate oxidoreductase subunit IorA |
|  | RS_RS19235 | -1.56 | oxidoreductase |
|  | RS_RS12165 | 1.50 | oxidoreductase |
|  | RS_RS21480 | 1.57 | LLM class oxidoreductase |
|  | RS_RS23455 | 1.70 | amine oxidase |
|  | RS_RS23585 | 1.70 | amine oxidase |
|  | RS_RS24655 | 1.98 | bb3-type cytochrome oxidase subunit IV |
|  | RS_RS20850 | -4.59 | aminoacyl-tRNA synthet |
| Lipid metabolism | RS_RS15300 | -1.55 | glycerol-3-phosphate dehydrogenase |
|  | RS_RS20870 | -4.41 | beta-ketoacyl-ACP synthase |
|  | RS_RS20900 | -4.51 | acyl-CoA desaturase |
|  | *glpK* | -1.58 | glycerol kinase |
|  | RS_RS08885 | -1.86 | 3-hydroxyacyl-CoA dehydrogenase |
|  | RS_RS15725 | -1.86 | zinc-dependent alcohol dehydrogenase |
|  | RS_RS19230 | -2.23 | alcohol dehydrogenase |
|  | RS_RS24750 | 3.36 | aldehyde dehydrogenase family protein |
|  | RS_RS08835 | -2.35 | long-chain-fatty-acid--CoA ligase |
|  | RS_RS03920 | -1.61 | long-chain-fatty-acid--CoA ligase |
|  | RS_RS20860 | -5.27 | omega-3 fatty acid desaturase |
|  | RS_RS12705 | -3.00 | 3-hydroxyacyl-CoA dehydrogenase |
|  | RS_RS06850 | -2.88 | acyl-CoA-binding protein |
|  | RS_RS03155 | 1.81 | lipoprotein |
| Transporter | RS_RS02740 | -3.28 | MFS transporter |
|  | RS_RS23960 | -3.24 | MFS transporter |
|  | RS_RS20595 | -2.49 | MFS transporter |
|  | RS_RS09365 | -2.21 | multidrug transporter |
|  | RS_RS24390 | -1.92 | nitrate ABC transporter substrate-binding protein |
|  | RS_RS10170 | -1.86 | amino acid transporter |
|  | RS_RS00920 | -1.75 | transporter |
|  | RS_RS19740 | -1.71 | MFS transporter |
|  | RS_RS22495 | -1.68 | RND transporter |
|  | RS_RS22490 | -1.66 | multidrug efflux RND transporter permease subunit |
|  | RS_RS22485 | -1.66 | MexE family multidrug efflux RND transporter periplasmic adaptor subunit |
|  | RS_RS24385 | -1.63 | ABC transporter permease |
|  | RS_RS24380 | -1.61 | sulfonate ABC transporter ATP-binding lipoprotein |
|  | RS_RS10870 | -1.56 | C4-dicarboxylate ABC transporter |
|  | RS_RS08865 | -2.57 | transposase |
|  | RS_RS26545 | 1.53 | remnant of isrso16-transposase orfb protein |
|  | RS_RS25305 | 1.79 | probable remnant of a transposase protein |
|  | RS_RS26600 | 1.88 | IS3 family transposase ISRso11 |
|  | RS_RS26380 | 2.37 | transposase |
|  | RS_RS26415 | 2.37 | transposase |
|  | RS_RS15955 | -1.70 | GNAT family N-acetyltransferase |
|  | RS_RS21640 | 1.96 | methyltransferase |
|  | RS_RS14345 | 1.50 | ABC transporter permease |
|  | RS_RS16050 | 1.50 | transporter |
|  | RS_RS17230 | 1.52 | MFS transporter |
|  | RS_RS22405 | 1.55 | peptide transporter |
|  | RS_RS17565 | 1.56 | MFS transporter |
|  | RS_RS20325 | 1.68 | RND transporter |
|  | RS_RS17330 | -1.55 | DNA-binding protein |
|  | RS_RS11650 | 1.85 | MFS transporter |
|  | RS_RS23525 | 1.87 | RhtB family transporter |
|  | RS_RS23655 | 1.88 | RhtB family transporter |
|  | RS_RS16530 | 2.23 | potassium transporter Kef |
|  | RS_RS17320 | 2.97 | manganese transporter |
|  | RS_RS19560 | 3.32 | MFS transporter |
|  | RS_RS01005 | -3.71 | hypothetical h+-transporting two-sector atpase, gamma subunit; protein |
|  | *corA* | 1.74 | magnesium and cobalt transport protein CorA |
| Regulator | RS_RS21775 | -3.57 | Crp/Fnr family transcriptional regulator |
|  | RS_RS05515 | -3.35 | LysR family transcriptional regulator |
|  | RS_RS02690 | -3.01 | transcription regulator protein |
|  | RS_RS06490 | -2.70 | MarR family transcriptional regulator |
|  | RS_RS02340 | -2.35 | TetR family transcriptional regulator |
|  | RS_RS14005 | -2.29 | AsnC family transcriptional regulator |
|  | RS_RS12710 | -1.93 | probable transcription regulator protein |
|  | RS_RS13815 | -1.90 | LysR family transcriptional regulator |
|  | RS_RS22475 | -1.84 | LysR family transcriptional regulator |
|  | RS_RS05425 | -1.80 | LysR family transcriptional regulator |
|  | RS_RS10035 | -1.76 | probable transcription regulator protein |
|  | RS_RS23130 | -1.73 | transcriptional regulator |
|  | RS_RS03815 | -1.70 | IclR family transcriptional regulator |
|  | RS_RS09350 | -1.67 | MarR family transcriptional regulator |
|  | RS_RS06905 | -1.63 | MurR/RpiR family transcriptional regulator |
|  | RS_RS16695 | -1.58 | LysR family transcriptional regulator |
|  | RS_RS00055 | -1.57 | TetR family transcriptional regulator |
|  | RS_RS21630 | -1.56 | LysR family transcriptional regulator |
|  | RS_RS21740 | -1.55 | nitric oxide reductase transcription regulator |
|  | RS_RS19830 | -1.50 | transcriptional regulator |
|  | RS_RS01430 | 1.50 | response regulator |
|  | RS_RS25105 | 1.54 | sigma-54-dependent Fis family transcriptional regulator |
|  | RS_RS02765 | 1.57 | regulatory protein RecX |
|  | RS_RS22565 | 1.58 | transcriptional regulator |
|  | RS_RS05345 | 1.59 | probable two-component system response regulator transcription regulator protein |
|  | RS_RS22745 | 1.68 | transcription regulator protein |
|  | RS_RS25110 | 1.82 | sigma-54-dependent Fis family transcriptional regulator |
|  | RS_RS15820 | 1.98 | transcriptional regulator |
|  | RS_RS23510 | 1.99 | transcriptional regulator |
|  | RS_RS23640 | 1.99 | transcriptional regulator |
|  | RS_RS05945 | 2.05 | Fis family transcriptional regulator |
|  | RS_RS15885 | 2.18 | DNA-binding response regulator |
|  | RS_RS09495 | 2.26 | LysR family transcriptional regulator |
|  | RS_RS08435 | 2.60 | Phage regulatory protein CII (CP76) |
|  | RS_RS25480 | 2.64 | prophage regulatory protein |
| Signal transduction | RS_RS21840 | -5.57 | histidine kinase |
|  | RS_RS21845 | -2.94 | DNA-binding response regulator |
|  | RS_RS26305 | 1.89 | Sensor signal transduction histidine kinase |
|  | RS_RS18210 | 2.66 | two-component sensor histidine kinase |
|  | *egtD* | 1.55 | L-histidine N(alpha)-methyltransferase |
|  | RS_RS17955 | 1.74 | protein prenyltransferase |
|  | RS_RS20880 | -4.81 | carbamoyltransferase |
|  | RS_RS19025 | -1.56 | mannosyltransferase |
|  | RS_RS00905 | -4.52 | histidine kinase |
|  | RS_RS25120 | 2.37 | RNA polymerase sigma-54 factor |
|  | RS_RS13655 | -2.24 | TonB-dependent siderophore receptor |
| Metabolism of cofactors and vitamins | *hemN* | -7.36 | coproporphyrinogen III oxidase |
|  | RS_RS09190 | 2.78 | probable maleate cis-trans isomerase protein |
|  | RS_RS00555 | 1.60 | phosphomethylpyrimidine synthase |
|  | RS_RS17975 | 2.24 | NIFS-like protein |
| Replication and repair | *recA* | 1.80 | DNA recombination/repair protein RecA |
|  | RS_RS18345 | 2.30 | transcriptional repressor |
|  | RS_RS20930 | 2.22 | cell division protein |
|  | RS_RS01575 | -6.16 | twin-arginine translocation protein |
| Metabolism of other amino acids | RS_RS10110 | -1.85 | glutathione S-transferase |
|  | RS_RS16580 | 2.14 | glutathione S-transferase |
|  | *ggt* | -2.46 | gamma-glutamyltransferase |
|  | RS_RS00415 | -1.80 | aspartate aminotransferase family protein |
|  | RS_RS06940 | -1.77 | D-aminopeptidase |
|  | RS_RS19110 | 2.66 | peptidase |
|  | RS_RS21065 | -1.73 | spermidine synthase |
|  | RS_RS19555 | 2.74 | methionine biosynthesis protein MetW |
|  | RS_RS19485 | 2.14 | amidinotransferase |
|  | RS_RS00420 | -2.07 | 3-keto-5-aminohexanoate cleavage enzyme |
|  | RS_RS07510 | -2.29 | Valine--pyruvate transaminase |
| Xenobiotics biodegradation and metabolism | RS_RS20525 | 2.04 | cytochrome P450 |
|  | RS_RS08945 | -2.69 | 4-hydroxybutyrate dehydrogenase |
|  | RS_RS23460 | 1.86 | carboxymethylenebutenolidase |
|  | RS_RS10890 | -1.86 | allophanate hydrolase |
|  | RS_RS23590 | 1.86 | carboxymethylenebutenolidase |
|  | RS_RS09535 | 1.62 | alkylhydroperoxidase |
|  | RS_RS02215 | 2.04 | acetate kinase |
|  | RS_RS01720 | 1.92 | bacterioferritin-associated ferredoxin protein |
|  | *pobA* | -3.06 | 4-hydroxybenzoate 3-monooxygenase |
|  | RS_RS21405 | 1.55 | 4-oxalocrotonate decarboxylase |
|  | RS_RS21385 | 1.95 | 2,3-dihydroxy-p-cumate-3,4-dioxygenase (CmtC) (plasmid) |
| Nucleotide metabolism | RS_RS14025 | 1.53 | ribonucleotide-diphosphate reductase subunit alpha |
|  | RS_RS21760 | -2.37 | anaerobic ribonucleoside triphosphate reductase |
|  | RS_RS01000 | -3.71 | phosphoribosylpyrophosphate synthetase |
|  | RS_RS16480 | 1.80 | ATP-dependent DNA helicase Rep |
|  | RS_RS20940 | 1.88 | error-prone DNA polymerase |
|  | RS_RS20935 | 2.81 | DNA polymerase IV protein |
|  | RS_RS04735 | 2.55 | ATP-dependent RNA helicase |
|  | RS_RS18505 | 12.36 | GGDEF domain-containing protein |
|  | RS_RS03750 | 1.93 | endonuclease |
|  | RS_RS19210 | 1.69 | phosphoribosyltransferase |
|  | RS_RS04200 | -3.40 | RNA polymerase subunit sigma |
|  | RS_RS07360 | -2.03 | Nucleoside-diphosphate-sugar epimerase |
|  | RS_RS20480 | 2.58 | RtcB family protein |
| Biosynthesis of other secondary metabolites | RS_RS26360 | 2.42 | hypothethical protein (plasmid) |
|  | RS_RS15890 | 2.33 | hemagglutinin |
|  | RS_RS02540 | 4.82 | bifunctional diguanylate cyclase/phosphodiesterase |
|  | RS_RS18030 | 2.12 | hemagglutinin |
|  | RS_RS17980 | 2.13 | SAM-dependent methyltransferase |
|  | RS_RS07525 | 1.78 | FkbM family methyltransferase |
|  | RS_RS20835 | -3.33 | N-acylhomoserine lactone synthase |
|  | RS_RS03905 | -2.73 | hemerythrin |
|  | RS_RS16910 | 1.91 | Panthothenate synthetase |
|  | *acpP* | 1.53 | acyl carrier protein |
|  | RS_RS20895 | -5.12 | acyl carrier protein |
|  | RS_RS21440 | 1.53 | hydratase |
|  | RS_RS12725 | -3.31 | bile acid:sodium symporter |
|  | *prpF* | -1.72 | 3-methylitaconate isomerase (plasmid) |
| Metabolism of terpenoids and polyketides | RS_RS12980 | -1.50 | limonene-1,2-epoxide hydrolase |
|  | RS_RS17310 | 2.70 | porin |
|  | RS_RS20570 | -2.45 | porin |
|  | RS_RS24940 | -2.17 | porin |
|  | RS_RS18245 | -1.92 | feruloyl-CoA synthase |
| Folding, sorting and degradation | *groL* | -1.86 | molecular chaperone GroEL |
|  | RS_RS00995 | -5.57 | MBL fold metallo-hydrolase |
|  | RS_RS11100 | -5.50 | MBL fold metallo-hydrolase |
|  | RS_RS19960 | 1.64 | MBL fold metallo-hydrolase |
|  | RS_RS02965 | -1.87 | phosphohydrolase |
| Hypothetical protein and others | RS_RS21730 | 2.24 | hypothetical protein |
|  | RS_RS25945 | -2.52 | hypothetical protein |
|  | RS_RS26095 | 1.70 | hypothetical protein |
|  | RS_RS04775 | 1.72 | hypothetical protein |
|  | RS_RS25225 | 2.10 | hypothetical protein |
|  | RS_RS25400 | 2.87 | hypothetical protein |
|  | RS_RS24550 | 3.35 | hypothetical protein |
|  | RS_RS26475 | 3.42 | hypothetical protein |
|  | RS_RS02975 | 3.71 | hypothetical protein |
|  | RS_RS22740 | 3.80 | conserved hypothethical protein (plasmid) |
|  | RS_RS19565 | 3.51 | conserved hypothethical protein (plasmid) |
|  | RS_RS16215 | 3.55 | conserved hypothethical protein |
|  | RS_RS16220 | 12.03 | Conserved hypothethical protein, DNA-binding domain |
|  | RS_RS20885 | -5.14 | conserved hypothethical protein (plasmid) |
|  | RS_RS20865 | -4.94 | conserved hypothethical protein (plasmid) |
|  | RS_RS20905 | -2.72 | conserved hypothethical protein (plasmid) |
|  | RS_RS19245 | -2.31 | conserved hypothethical protein (plasmid) |
|  | RS_RS20630 | -1.71 | conserved hypothethical protein (plasmid) |
|  | RS_RS20635 | -1.65 | conserved hypothethical protein (plasmid) |
|  | RS_RS25495 | -1.58 | conserved hypothethical protein (plasmid) |
|  | RS_RS17965 | 1.59 | conserved hypothethical protein (plasmid) |
|  | RS_RS20195 | 1.60 | conserved hypothethical protein (plasmid) |
|  | RS_RS21725 | 1.66 | Conserved hypothethical protein |
|  | RS_RS20035 | 2.01 | conserved hypothethical protein, UCP019302 (plasmid) |
|  | RS_RS19220 | 2.16 | conserved hypothethical protein (plasmid) |
|  | RS_RS25135 | 2.33 | conserved hypothethical protein (plasmid) |
|  | RS_RS17970 | 2.56 | Conserved hypothethical protein |
|  | RS_RS02985 | 2.64 | Conserved hypothethical protein |
|  | RS_RS19545 | 2.79 | conserved hypothethical protein |
|  | RS_RS19550 | 2.80 | conserved hypothethical protein (fragment) |
|  | RS_RS18350 | 2.83 | conserved hypothethical protein (plasmid) |
|  | RS_RS16920 | 2.87 | conserved hypothethical protein |
|  | RS_RS01105 | 3.14 | conserved hypothethical protein |
|  | RS_RS19115 | 3.23 | conserved hypothethical protein |
|  | RS_RS18945 | 3.40 | conserved hypothethical protein |
|  | RS_RS19570 | 3.61 | Conserved hypothethical protein |
|  | RS_RS17325 | 2.75 | GALA protein |
|  | RS_RS17400 | 2.15 | Uncharacterised protein |
|  | RS_RS16730 | -3.09 | Uncharacterised protein |
|  | RS_RS25125 | 2.09 | DUF2334 domain-containing protein |
|  | RS_RS00900 | -12.99 | DUF2892 domain-containing protein |
|  | RS_RS03840 | -5.82 | DUF3079 domain-containing protein |
|  | RS_RS02970 | -1.89 | conserved protein of unknown function |
|  | RS_RS25420 | 1.59 | conserved protein of unknown function |
|  | RS_RS00955 | -4.07 | conserved protein of unknown function |
|  | RS_RS07520 | 2.02 | TPR domain protein |
|  | RS_RS04415 | 1.95 | HrgA protein |
|  | RS_RS02915 | 1.86 | probable avrd-related protein |
|  | RS_RS08485 | 1.83 | phage portal protein |
|  | RS_RS20330 | 1.81 | acriflavine resistance protein B |
|  | RS_RS25595 | 1.60 | Hypothethical protein |
|  | RS_RS16195 | 1.71 | Uncharacterized conserved protein |
|  | RS_RS02980 | 1.59 | TonB-dependent receptor |
|  | RS_RS23530 | 1.58 | RidA family protein |
|  | RS_RS23660 | 1.58 | RidA family protein |
|  | RS_RS16205 | 1.53 | phage related protein |
|  | RS_RS04170 | -1.74 | phage related protein |
|  | RS_RS25630 | -1.72 | histone |
|  | RS_RS09685 | 1.50 | phage tail protein |
|  | RS_RS07590 | -3.55 | DUF1488 domain-containing protein |
|  | RS_RS23950 | -3.62 | FlxA-like protein |
|  | RS_RS07560 | -6.69 | universal stress protein UspA |
|  | RS_RS00885 | -5.89 | universal stress protein UspA |
|  | RS_RS00990 | -5.46 | heat-shock protein Hsp20 |
|  | RS_RS20590 | -5.30 | radical SAM protein |
|  | RS_RS21785 | -4.81 | SCP2 domain-containing protein |
|  | RS_RS00910 | -3.66 | universal stress protein UspA |
|  | RS_RS21225 | -3.58 | HrpX protein |
|  | RS_RS00915 | -3.31 | universal stress protein UspA |
|  | RS_RS21330 | -2.97 | protein PopA1 |
|  | RS_RS21325 | -2.13 | protein PopB |
|  | RS_RS10885 | -2.57 | LamB/YcsF family protein |
|  | RS_RS24350 | -2.00 | SCO family protein |
|  | RS_RS18675 | -1.96 | universal stress protein UspA |
|  | RS_RS24625 | -1.88 | universal stress protein UspA |
|  | RS_RS19310 | -1.86 | Immunity protein 58 |
|  | RS_RS17030 | -1.51 | flavohemoprotein |
|  | RS_RS03225 | -1.93 | molecular chaperone GroES |
|  | RS_RS18100 | -1.51 | disulfide bond formation protein B |
|  | RS_RS19240 | -1.76 | anti-ECFsigma factor, ChrR |
