# Supplementary material for: Anthranilic acid from Ralstonia solanacearum plays dual roles in intraspecies signalling and inter-kingdom communication
Source: ISME J. 2020 May 26;14(9):2248–60. doi: 10.1038/s41396-020-0682-7 (PMC7608240; doi:10.1038/s41396-020-0682-7)
Supplement: Supplementary file 27 — Supplementary Table 5 [file 41396_2020_682_MOESM27_ESM.docx]

**Supplementary Table 5** Analysis of the homologues of *trpE* and *trpG* in various bacterial species

| Bacteria | Strain | *trpE* homologue  Accession No. | | *trpE* homologue  Identity (%) | *trpG* homologue  Accession No. | *trpG* homologue  Identity (%) | |
| --- | --- | --- | --- | --- | --- | --- | --- |
| ***Ralstonia*** |  | |  |  |  |  |  |
| *R. solanacearum* | GMI1000 | | WP_011002787 | 100 | WP_011002788 | 100 |  |
| *R. syzygii* | R24 | | CCA85208 | 97 | CCA85209 | 93 |  |
| *R. mannitolilytica* | SN83A39 | | ANA33024 | 91 | ANA33025 | 90 |  |
| *R. insidiosa* | ATCC 49129 | | ANJ73736 | 90 | ANJ73737 | 89 |  |
| *R. pickettii* | 12J | | ACD28246 | 90 | ACD28247 | 90 |  |
| *R. eutropha* | H16 | | CAJ94388 | 85 | CAJ94389 | 83 |  |
| ***Cupriavidus*** |  | |  |  |  |  |  |
| *C. nantongensis* | X1 | | AMR79934 | 86 | AMR79935 | 84 |  |
| *C. necator* | NH9 | | AQV95728 | 86 | AQV95729 | 83 |  |
| *C. gilardii* | CR3 | | ALD91649 | 86 | CP010516 | 82 |  |
| *C. basilensis* | 4G11 | | AJG21086 | 85 | CP010536 | 82 |  |
| *C. taiwanensis* | LMG19424 | | CAQ70700 | 85 | CU633749 | 82 |  |
| ***Burkholderia*** |  | |  |  |  |  |  |
| *B. cenocepacia* | H111 | | CDN58912 | 88 | CDN58911 | 81 |  |
| *B. glumae* | PG1 | | AJK44965 | 87 | AJK44950 | 87 |  |
| *B. plantarii* | ATCC 43733 | | ALK29242 | 87 | ALK29227 | 87 |  |
| *B. thailandensis* | MSMB121 | | AGK46841 | 80 | CP004095 | 81 |  |
| *B. stagnalis* | MSMB735WGS | | CP013459 | 80 | CP013459 | 81 |  |
| *B. pseudomallei* | 2008724758 | | CP018383 | 80 | CP018383 | 80 |  |
| *B. mallei* | 2002734306 | | CP009708 | 80 | CP009708 | 81 |  |
| ***Pandoraea*** |  | |  |  |  |  |  |
| *P. pulmonicola* | DSM 16583 | | CP010310 | 81 | CP010310 | 80 |  |
| *P. pnomenusa* | DSM 16536 | | CP009553 | 80 | CP009553 | 80 |  |
| *P. mesoacidophila* | ATCC 31433 | | CP020737 | 80 | CP020737 | 81 |  |
| *P. oxalativorans* | DSM 23570 | | CP011253 | 79 | CP011253 | 77 |  |
| *P. faecigallinarum* | DSM 23572 | | CP011807 | 79 | CP011807 | 79 |  |
| *P. sputorum* | DSM 21091 | | CP010431 | 79 | CP010431 | 78 |  |
| *P. vervacti* | NS15 | | CP010897 | 79 | CP010897 | 78 |  |
| *P. norimbergensis* | DSM 11628 | | CP013480 | 78 | CP013480 | 79 |  |
| *P. apista* | DSM 16535 | | CP013481 | 78 | CP013481 | 78 |  |
| ***Collimonas*** |  | |  |  |  |  |  |
| *C. fungivorans* | Ter331 | | CP002745 | 79 |  |  |  |
| *C. pratensis* | Ter291 | | CP013236 | 78 |  |  |  |
| *C. arenae* | Ter282 | | CP013235 | 77 |  |  |  |
| ***Achromobacter*** |  | |  |  |  |  |  |
| *A. xylosoxidans* | FDAARGOS_147 | | CP014060 | 78 | CP014060 | 76 |  |
| *A.* *sp.* | MFA1 R4 | | LT707065 | 77 |  |  |  |
| *A.* *denitrificans* | USDA-ARS-USMARC-56712 | | CP013923 | 77 | CP013923 | 76 |  |
| ***Comamonas*** |  | |  |  |  |  |  |
| *C. serinivorans* | DSM 26136 | | CP021455 | 77 |  |  |  |
| *C. cancerogenus* | ATCC35316 | | EFC53626 | 38 |  |  |  |
| ***Bordetella*** |  | |  |  |  |  |  |
| *B. pseudohinzii* | HI4681 | | CP016440 | 78 |  |  |  |
| *B. hinzii* | H568 | | CP012077 | 78 |  |  |  |
| *B. bronchialis* | AU3182 | | CP016170 | 77 |  |  |  |
| *B.* *bronchiseptica* | I328 | | CP016431 | 77 |  |  |  |
| *B.* *pertussis* | J446 | | CP017403 | 77 |  |  |  |
| *B.* *petrii* | DSM 12804 | | AM902716 | 76 |  |  |  |
| *B.* *parapertussis* | Bpp5 | | HE965803 | 76 |  |  |  |
| *B.* *flabilis* | AU10664 | | CP016172 | 76 |  |  |  |
| ***Others*** |  | |  |  |  |  |  |
| *Janthinobacterium sp.* | LM6 | | CP019510 | 77 |  |  |  |
| *Castellaniella defragrans* | 65Phen | | HG916765 | 76 | AEX50510 | 58 |  |
| *Herbaspirillum hiltneri* | N3 | | CP011409 | 79 | ALB66947 | 56 |  |
| *Pseudomonas mesoacidophila* | ATCC 31433 | | CP020737 | 80 | CP020737 | 81 |  |
| *Leptothrix cholodnii* | SP-6 | | CP001013 | 78 | CP001013 | 77 |  |
